# Adverse Outcomes and Associated Factors Among Children and Youths With Diabetes Mellitus in East Africa: A Systematic Review and Meta‐Analysis

**DOI:** 10.1111/jan.70124

**Published:** 2025-08-04

**Authors:** Chalie Marew Tiruneh, Marilyn Cruickshank, Muhammad Chutiyami, Lin Perry

**Affiliations:** ^1^ School of Nursing and Midwifery, Faculty of Health University of Technology Sydney Ultimo New South Wales Australia; ^2^ Department of Pediatrics and Child Health Nursing, College of Health Sciences Debre Tabor University Amhara Ethiopia; ^3^ Sydney Children's Hospitals Network Sydney New South Wales Australia

**Keywords:** adolescents, children, diabetes complications, diabetes management, East Africa, health outcomes, nursing, quality of life, type 1 diabetes mellitus

## Abstract

**Aim:**

To identify adverse outcomes and factors associated with diabetes mellitus among children and youths in East Africa.

**Design:**

This review was conducted following the Preferred Reporting Items for Systematic Reviews and Meta‐Analyses guidelines.

**Data Sources:**

No date restrictions were applied to searches of the Ovid MEDLINE, Embase, PubMed, CINAHL, Scopus, Web of Science, Cochrane Library databases and Google Scholar. The review identified and included literature published between 2007 and 2024.

**Methods:**

Independent reviewers conducted study selection, data extraction, and quality assessment. Data were organised in Microsoft Excel, detailing study characteristics, demographics, exposures, and outcomes. Narrative synthesis summarised the data, while meta‐analysis yielded pooled proportions.

**Results:**

From 3797 publications, 30 studies involving 6109 children and youths with type 1 diabetes were included. Findings revealed that between 39.3% and 99% did not achieve target glycaemic levels. Diabetic ketoacidosis at diagnosis ranged from 35.8% to 78.7%. Pooled estimates indicated mortality in 6.47%, nephropathy in 15.66%, and retinopathy in 27.49% of the cases. Other complications included decreased health‐related quality of life, lipodystrophy, psychiatric disorders, and stunting.

**Conclusion:**

This review highlights the need for context‐specific, personalised diabetes care for children and youths in East Africa. It underscores the need for healthcare professionals, particularly nurse diabetes educators, to provide personalised, holistic care and education. Policies that strengthen health systems, expand health insurance, and improve access to care are critical priorities to improve outcomes for these populations.

**Impact:**

This study provides new information on diabetes‐related complications and management challenges among children and youths in East Africa. Findings flag the urgent need for integrated care, standardised diagnostic criteria, and improved access to resources, with implications for healthcare providers, policymakers, and researchers to enhance health outcomes and quality of life.

**Patient or Public Contribution:**

This study did not include patient or public involvement in its design, conduct, or reporting.

**Abbreviations**
CI: confidence interval
CINAHL: cumulative index to nursing and allied health literature
DKA: diabetic ketoacidosis
DM: diabetes mellitus
DQOLY‐SF: diabetes quality of life for youth (short form)
HbA1c: haemoglobin A1c
HRQoL: health‐related quality‐of‐life
JBI: Joanna Briggs institute
LMICs: low‐ and middle‐income countries
MODY: maturity‐onset diabetes of the young
NAFLD: non‐alcoholic fatty liver disease
OR: odds ratio
PedsQL: paediatric quality of life inventory
PEO: population, exposure, and outcome
POR: pooled odds ratio
PRISMA: preferred reporting items for systematic reviews and meta‐analysis
SD: standard deviation
STATA: statistical analysis software
T1DM: type 1 diabetes mellitus T2DM

## 1 Introduction

Diabetes mellitus is a chronic, life‐limiting condition that is becoming more common worldwide, including in low‐ and middle‐income countries (LMICs) such as those in East Africa (Libman et al. 2022). Globally, an estimated 537 million adults aged 20–79 years are currently living with diabetes, representing 10.5% of the world's population in this age group in 2021 (International Diabetes Federation 2021). Diabetes is ranked among the top 10 leading causes of disability and in 2020/2021 was the 7th leading cause of global Disability‐Adjusted Life Years (DALYs), responsible for 76.5 million and 78.9 million DALYs, respectively (Alene et al. 2024).

Children, adolescents, and youths can be diagnosed with type 1 diabetes (T1DM), type 2 diabetes (T2DM), gestational diabetes mellitus (GDM) in adolescent pregnancy (though uncommon), or other forms such as neonatal diabetes, monogenic diabetes (1%–6% of autoantibody‐negative individuals) (Libman et al. 2022), and steroid‐induced diabetes (Graham Ogle et al. 2017). T1DM affects individuals of all ages, including children and youths. Previously considered a condition primarily affecting adults, the number of children and youths with T2DM has been rising in recent years, and this pattern is expected to persist (Lawrence et al. 2021; Libman et al. 2022).

T1DM and T2DM are becoming increasingly common in children and youths, and their clinical and public health significance is increasingly recognised by clinicians and researchers (Al‐Kandari et al. 2020; Lawrence et al. 2021). However, the prevalence of T1DM is higher and has a significantly greater impact compared to T2DM (International Diabetes Federation 2021). Diabetes rates and growth in these rates vary globally, with certain regions experiencing particularly alarming increases. In 2021, an estimated 108,300 children and adolescents under 15 years were newly diagnosed with T1DM globally, with 651,700 living with the condition (Libman et al. 2022). In East Africa, T1DM cases in those under 19 increased five‐fold from 2011 to 2021, from 4 to nearly 20 per 1000 children, alongside worsening health outcomes (Kidane et al. 2023).

Increasing rates of diabetes are being accompanied by increases in children's and youths' risk of early complications, comorbidities, and mortality, attributed to multiple factors including physiological, emotional, and social changes during these developmental stages. This increase is further exacerbated by barriers such as delayed diagnosis, which may result from limited access to healthcare, a lack of awareness about diabetes, and inadequate care in resource‐limited settings (Glaser et al. 2022; Graham Ogle et al. 2017; Nam Hoon 2019). Onset of complications may be delayed or deterred by optimising glycaemic control, and evidence has shown that regular contact with diabetes care providers supports this (Owusu and Doku 2024). Recent molecular evidence also suggests a role for the anti‐ageing gene Sirtuin 1 in diabetes prevention and management, particularly in children. For the future, early assessment of plasma Sirtuin 1 levels may provide prognostic value, and its activation through nutritional interventions might improve metabolic regulation, potentially reducing complications (Martins 2018a, 2018b).

Despite the growing burden, diabetes in children and youths has received limited attention in East Africa in terms of research, funding, and health system response. Whilst some primary studies have been conducted on glycaemic levels and outcomes of diabetes mellitus, no systematic or scoping review has synthesised regional evidence on adverse outcomes or quality of life. As a result, the scope and scale of evidence on these issues remain poorly understood. To rectify this gap, this study aimed to examine evidence for adverse outcomes associated with T1DM and T2DM and to identify information gaps about the acute and chronic complications and health‐related quality of life among children and youths living with the conditions in East Africa. Such findings are essential to inform future research, policy, and clinical practice, including nursing care strategies aimed at improving health outcomes and reducing healthcare costs in these populations.

Questions addressed by this review were therefore:

- What adverse outcomes are associated with T1DM and T2DM among children and youths in East Africa?
- What factors are associated with adverse outcomes among children and youths with T1DM and T2DM in East Africa?

## 2 Methods

### 2.1 Design and Protocol

A systematic review protocol was developed and registered under the reference number CRD42023485671 in the PROSPERO database. The Preferred Reporting Items for Systematic Reviews and Meta‐Analyses (PRISMA) guideline (Page et al. 2021) requirements were followed in this review report.

### 2.2 Search Methods

The search strategy was developed using the Population, Exposure, and Outcome (PEO) framework. The population (P) included children and youths aged ≤ 19 years with diabetes mellitus in East Africa. The exposure (E) of interest was a diagnosis of type 1 diabetes mellitus (T1DM) or type 2 diabetes mellitus (T2DM). The outcomes (O) focused on adverse outcomes and complications, including poor glycaemic control, diabetic ketoacidosis (DKA), hyperglycaemic hyperosmolar state, hypoglycaemia, retinopathy, nephropathy, neuropathy, cardiovascular complications, psychological effects (such as stress, anxiety, and depression), gangrene, developmental delays, poor school performance, lipodystrophy, and mortality. The search was conducted using a combination of relevant MESH terms, keywords, and synonyms in titles or abstracts, with Boolean operators and wildcards applied as appropriate for each database (Table S1).

### 2.3 Inclusion and/or Exclusion Criteria

#### 2.3.1 Inclusion Criteria

##### 2.3.1.1 Study Setting

Health facility‐based studies were selected (for consistency of the data), conducted in East African countries classified according to the United Nations Statistics Division. This classification includes 17 countries, one de facto state, and four overseas departments (Table S1) (United Nations 2023).

##### 2.3.1.2 Study Population

Studies were required to have been conducted among children and youths aged ≤ 19 years with T1DM or T2DM.

##### 2.3.1.3 Study Design

Longitudinal and cross‐sectional studies were included, recruiting retrospectively or prospectively and published in English.

#### 2.3.2 Exclusion Criteria

Studies employing designs including secondary data reviews, case reports, case series, qualitative studies, or opinion papers were excluded. However, reference lists of reviews were searched for eligible primary sources.

##### 2.3.2.1 Study Population

Studies were excluded where participants had forms of diabetes other than T1DM or T2DM; where studies included both adult and child populations and did not supply separate analyses for children and youths or were not clear about participants' ages.

### 2.4 Data Sources

Searches were conducted through the Ovid MEDLINE, Excerpta Medica (Embase), PubMed, CINAHL, Scopus, and Web of Science databases, and the Cochrane Library by the first author (CMT). The studies included in this review were published between 2007 and 2024, although no date restrictions were applied during the database searches. The search was conducted from October 4 to 28, 2023, and was updated from January 18 to 20, 2025. Additional searches were conducted using Google Scholar, screening the first five pages to identify relevant studies published during the same period.

### 2.5 Selection Process

After searching, the results were imported into Covidence for screening. Duplicates were removed using Covidence's automated system and manual checks. The first author (CMT) screened titles and abstracts against pre‐specified inclusion and exclusion criteria, followed by full‐text screening of eligible studies. To ensure rigour, 10% of the records at each stage were independently reviewed by a second reviewer (MC, CM, or LP), with discrepancies resolved through discussion.

### 2.6 Data Collection Process

A Microsoft Excel spreadsheet was created to collect study characteristics, participant characteristics, exposure, outcomes, and other relevant data from the included studies. The first author (CMT) extracted the data. Nine studies were randomly selected, and data were extracted independently by a second reviewer (MC, CM, or LP) to minimise bias and errors. In case of discrepancies, findings were compared, and consensus was reached through discussion or with a third reviewer (never required).

### 2.7 Data Items

The included studies reported outcomes of T1DM such as glycaemic level, DKA, mortality, health‐related quality of life, microvascular complications, psychiatric disorders, and length of hospital stay (Table S2). Dichotomous (presence/absence of DKA, meeting/not meeting recommended target glucose levels, mortality, presence of psychiatric disorder and microvascular complications) and continuous data (health‐related quality of life, HbA1c values, duration of hospital stay, number of diabetes complications) were reported. These outcomes were reported as proportions and mean (SD) values. The effect measure used for independent variables was the odds ratio (OR).

### 2.8 Risk of Bias Assessment

The quality of the included studies was critically evaluated by independent reviewers assessing methodological quality using the JBI appraisal tools for cross‐sectional or cohort studies (Moola et al. 2020). Within the process of quality assessment, all studies were evaluated by the first author (CMT), and a randomly selected sample of nine papers was independently critiqued by a second reviewer (MC, CM, or LP). Any disagreements were settled by double‐checking and discussing to a consensus with a third author (LP).

### 2.9 Data Synthesis and Analysis Methods

Data from the included studies were summarised using Microsoft Excel and synthesised narratively through texts, tables, and figures. Descriptive statistics were applied to highlight key study characteristics and findings.

A meta‐analysis of quantitative data relevant to outcomes of interest (where possible) was conducted using random‐effects meta‐analysis models. Pooled proportions and mean values were used to estimate T1DM outcomes, while odds ratios (OR) with 95% confidence intervals (CI) were calculated for associated factors.

Forest plots were generated to visually assess results and heterogeneity, a common challenge in meta‐analyses, representing the genuine variation in effect sizes attributed to intrinsic factors. Heterogeneity, representing the genuine variation in effect sizes attributed to intrinsic factors among the included studies (Borenstein et al. 2021), was evaluated using the Q test and quantified with I^2^ statistics. In the presence of heterogeneity, I^2^ was employed to measure its extent, while Galbraith plots were used to inspect the studies contributing to the heterogeneity visually. A leave‐one‐out meta‐analysis was conducted to investigate the source of heterogeneity. Publication bias, a potential challenge in conducting meta‐analysis (Rothstein et al. 2005), was checked by funnel plots produced for graphical diagnosis of small study effects. Additionally, the Begg and Egger regression tests with a *p*‐value < 0.05, along with nonparametric trim‐and‐fill analysis, were employed as the test statistics to assess the presence of publication bias (Sterne et al. 2001).

## 3 Results

### 3.1 Search Outcomes

**FIGURE 1:**
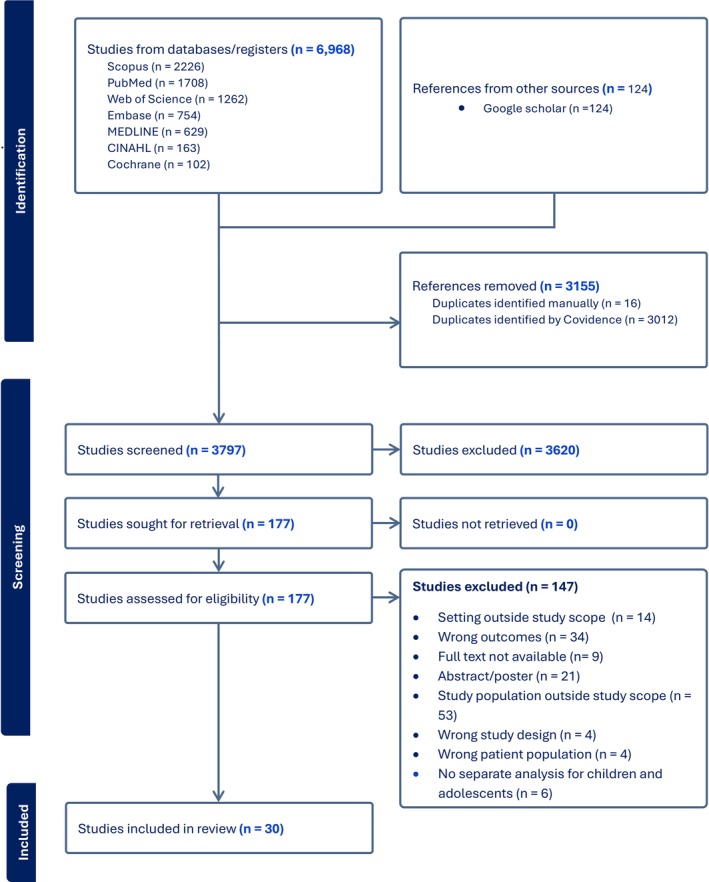
Preferred reporting items for systematic reviews and meta‐analyses flow diagram of the review.

After an initial and updated search, 6968 records were imported into Covidence. Following duplicate removal, 3797 studies underwent title and abstract screening, with 177 selected for full‐text review. Of these, 30 studies were eligible for data extraction (Figure 1). Despite a comprehensive search for both types of diabetes, the search results yielded studies exclusively focused on T1DM, and no studies addressing T2DM in this age group were found.

### 3.2 Risk of Bias Assessment

Included studies underwent assessment for methodological quality or risk of bias using the appropriate JBI tool (Moola et al. 2020). Studies were scored based on eight criteria for cross‐sectional studies and 11 criteria for cohort studies (Moola et al. 2020). Scores ranged from 4 to 7 for cross‐sectional studies (Table S3a) and from 7 to 10 for cohort studies (Table S3b). Despite limitations in certain components of the studies, including variability in outcome diagnostic criteria (especially for glycaemic level and DKA), limitations in the identification of confounding factors and oversight of important variables, most included studies scored higher than three‐fourths of the total possible quality score. Consequently, all 30 studies were included in the analysis (Tables S3a and S3b).

### 3.3 Characteristics of Included Studies

Of the 17 countries, four overseas departments, and one de facto state that were potential study sites, the included studies were from only six countries (Table 1). Ethiopia hosted the highest number of studies (*n* = 20, 66.7%) (Abrahim et al. 2023; AlehegnAwoke et al. 2024; Alemseged et al. 2024; Assefa et al. 2020; Atkilt et al. 2017; Bacha et al. 2022; Bekele et al. 2022; Eshetu et al. 2024; Gebeyehu et al. 2022; Girma et al. 2021; Habteyohans et al. 2023; Hadgu et al. 2019; Kidie et al. 2022; Kidie et al. 2021; Meseret 2021; Meseret et al. 2022; Shibeshi et al. 2022; Shibeshi et al. 2016; Shimelash et al. 2023; Tsadik et al. 2018), followed by Tanzania (*n* = 4, 13.3%) (Majaliwa et al. 2007; Msanga et al. 2020; Mukama et al. 2013; Noorani et al. 2016), Kenya (*n* = 2, 6.6%) (Musoma et al. 2020; Ngwiri et al. 2015), Rwanda (*n* = 2, 6.7%) (Gakuba 2016; Kayirangwa et al. 2018), Uganda (Lubwama 2022) and Malawi (Msekandiana et al. 2020) with *n* = 1 (3.3%) each.

**TABLE 1:** Methodological characteristics of included studies.

Author/year	Country	Study aim	Sample size, sampling technique; age, and sex distribution	Study design	Study period	Data collection method
Abrahim et al. (2023)	Ethiopia	To determine the glycaemic level and identify associated factors	A convenience sample; *n* = 158; mean (SD) age 12.05 ± 3.74 years (range 1.5–18 years); *n* = 86 (54.4%) males	Cross‐sectional study	July 10 to October 10, 2022.	Health record data were reviewed, and interviews; anthropometric assessments, and blood tests were conducted
AlehegnAwoke et al. (2024)	Ethiopia	To assess Prevalence and associated factors of psychiatric problems in children aged 6–18 years with type 1 diabetes mellitus	*n* = 206; convenient sampling technique; mean age of 12.8 years and SD of ±3.2; Males were higher (51.9%) than females (48.1%)	Cross‐sectional study design	February 1st to April 30th, 2022	interview using interviewer‐administered structured questionnaire
Alemseged et al. (2024)	Ethiopia	To determine the prevalence and identify associated factors of lipodystrophy in children and adolescents with type 1 diabetes mellitus	*n* = 122; convenient sampling; the median age was 13 years, distribution led by adolescents at 65 (53.3%) and children at 57 (46.7%); 63 (51.6%) males, M: F ratio of 1.07:1	Cross‐sectional study design	May 1 to July 31, 2020	Interview visual inspection and palpation of the injection site by paediatric resident physicians
Assefa et al. (2020)	Ethiopia	To assess the incidence and predictors of diabetic ketoacidosis	*n* = 354; simple random sampling; age less than 15 years and mean age ± SD = 8.21 ± 3.94 years; *n* = 159 (55.1%) were males	Retrospective follow‐up study	January 1, 2014, to January 1, 2019	Extraction of routine hospital health record data
Atkilt et al. (2017)	Ethiopia	To assess the prevalence and associated risk factors of DKA	*n* = 395; systematic random sampling; age < 12 years and mean age ± (SD) = 7.08 ± 3.8 years; *n* = 221 (55.9%) male	Cross‐sectional study	January 2009 to December 2014	Health record data were reviewed, and interviews for data not included in the patient card
Bacha et al. (2022)	Ethiopia	To determine the outcome of paediatric patients with DKA	*n* = 190; consecutive; Median age at presentation was 8 years; 42.6% (*n* = 81) were male	Cross‐sectional study	January 2013 to February 2017	Extraction of routine hospital health record data
Bekele et al. (2022)	Ethiopia	To determine the HRQoL and factors affecting it in children and adolescents with T1DM	*n* 379; simple random sampling; the mean age ± SD = 11.65 ± 3.56 years; *n* = 165 (43.5%) males	Cross‐sectional study	August 25, 2021, to September 25, 2021	An interview using the PedsQLTM 4.0, generic core scales, and anthropometry measurements
Eshetu et al. (2024)	Ethiopia	To assess the risk and predictors of chronic microvascular complications of type 1 diabetes mellitus among children with diabetes	*n* = 132; convenient sampling; 52 (41.9%) are at pubertal age (10–14 years); 66 (53%) were males	Ambi‐directional cohort study	September 10, 2021 to January 30, 2023	Interviewing the primary caregivers for children under 12 years old and Interviewing children above 12 years old. Extraction of routine hospital health record data
Gakuba (2016)	Rwanda	To determine factors that lead to improved QOL	*n* = 140; Age range 10–18 years; *n* = 61 (43.6%) males	Observational study using survey design	September 10th, 2015, to December 31, 2015	Interview; extraction of routine hospital data
Gebeyehu et al. (2022)	Ethiopia	To describe the clinical profiles and outcomes of children admitted with DKA	*n* = 175; systematic random sampling; *n* = 62, age range 8–15, mean 12.8 years; *n* = 105, 60% males	Retrospective cross‐sectional study	September 2015 to February 2020	Extraction of routine hospital data
Tsadik et al. (2018)	Ethiopia	To determine the magnitude of lipodystrophy, identify associated factors, and assess the impact of lipodystrophy on glycaemic level	*n* = 176; consecutive sampling; mean age ± SD = 11.36 ± 3.96, ranging 2–18 years; male to female ratio 1:1.05	Cross‐sectional study	April to July 2017	Extraction of routine hospital data; observation and palpation of lipodystrophy
Girma et al. (2021)	Ethiopia	To assess the health‐related Quality of life and its associated factors	*n* 229; systematic sampling; mean age ± SD = 12 ± 3; *n* = 127 (55.5%) were males	Cross‐sectional study	March 1 to May 30, 2018	An interview using the PedsQLTM 4.0, generic core scales, and anthropometry measurements
Habteyohans et al. (2023)	Ethiopia	To assess the magnitude of glucose levels where targets were unmet and associated factors	*n* = 231; consecutive sampling technique; age ± SD = 13 ± 4.9 years, ranged from 1 to 18 years *n* = 129 (55.8%) were males	Cross‐sectional study	November 15, 2022, to January 15, 2023	Interview and Extraction of routinely collected hospital health record data
Hadgu et al. (2019)	Ethiopia	To assess the prevalence and associated factors of DKA	*n* = 328, A convenient sample; age ranges between 3 months and 18 years; *n* = 143 (55.4%) males	Retrospective observational study	January 1, 2013, to December 30, 2017	Extraction of routinely collected hospital health record data
Kayirangwa et al. (2018)	Rwanda	To assess growth parameters of children with diabetes mellitus	*n* = 136; Ages ranged from 2 to 18 years old and the mean age ± SD = 14.3 ± 3.6); *n* = 54 (39.7%) were males	Cross‐sectional study	September–December 2015	Extraction of routine hospital health record data; Weight and height were plotted using WHO growth charts, weight for age and height for age, and HbA1c was measured by a trained technician
Kidie et al. (2021)	Ethiopia	To assess the frequency of DKA and its determinants	*n* = 354; simple random technique; age less than 15 years and the mean age ± SD = 8.21 ± 3.94 years; *n* = 159 (55.1%) were males	Retrospective follow‐up study	September 2015 to February 2018	Extraction of routine hospital health record data
Kidie et al. (2022)	Ethiopia	To assess the glycaemic level and associated factors	*n* = 389; simple random sampling; the mean age ± SD = 11 ± 4 years; included < 18 years of age; *n* = 174 (44.7%) were males	Cross‐sectional study	September 2015 to February 2018	Extraction of routine hospital health record data
Lubwama (2022)	Uganda	To determine the prevalence and the factors associated with microalbuminuria	*n* = 153 children and adolescents; consecutive sampling technique; Median age 8.4 y (6.12–12.09 y); *n* = 83 (54.2%) males	Cross‐sectional study	November 2020 to September 2021	Interview: Anthropometric and blood pressure assessments, and laboratory tests included urine albumin and urine creatinine, random blood glucose, HbA1c, serum urea, serum creatinine, and urine
Majaliwa et al. (2007)	Tanzania	To assess the glycaemic level and complications	*n* = 99; consecutive sampling technique; age range 5 to 18 years; *n* = 42 (43.3%) males	Cross‐sectional study	June 2005 to February 2006	Interview, anthropometric measurements, laboratory assessment; fundal ophthalmoscopy to assess diabetic‐related retinal damage
Meseret (2021)	Ethiopia	To determine the treatment outcome of children < 15 years admitted with DKA	*n* = 162, a consecutive sample of all children < 15 years with DKA; mean age ± SD = 8.1 ± 4.7 years; *n* = 83 (51.2%) males	Cross‐sectional study	January 1/2016 to February 30/2021	Extraction of routine hospital health record data
Meseret et al. (2022)	Ethiopia	To estimate time to target first optimal glycaemic achievement and identify predictors	*n* = 385 children < 15 years old; simple random sampling technique; Mean age ± SD was 8.9 ± 1.9 years; *n* = (53%) males	Retrospective follow‐up study	January 1, 2016 to February 30, 2021	Extraction of routine hospital health record data
Msanga et al. (2020)	Tanzania	To determine the prevalence of microvascular complications and associated Factors	All (*n* = 140); consecutive; age range 1–19 years; 50.3% of the males	Cross‐sectional study	Not clear	Interviews; anthropometric measurements and vital signs assessment, and laboratory assessment of serum creatinine
Msekandiana et al. (2020)	Malawi	To assess complication and glycaemic levels of adolescents with type 1 diabetes mellitus	*n* = 41; consecutive; 60.98%, *n* = 25 are in the age group of 11.5–15.5; 53.6%; *n* = 22 were males	Cross‐sectional study	October 2018 to June 2019	Extraction of routine hospital health record data; laboratory analysis of HA1c, Urine for microalbuminuria, and Slit Lamp ophthalmoscopy
Mukama et al. (2013)	Tanzania	To assess the impact of a 6‐month management and education programme on glycaemic level and acute complications	*n* = 81 consecutive; age less than 17 years; *n* = 50 (61.7%) were males	Prospective cohort study	August 2010 and April 2011	Interviews
Musoma et al. (2020)	Kenya	To determine the mortality and associated factors; to describe the average length of hospital stay	*n* = 159; consecutive; median age = 13 years (IQR 10–15), and *n* = 91 (57.2%) were females	Retrospective observational study	February 2013–February 2018	Extraction of routine hospital health record data
Ngwiri et al. (2015)	Kenya	To determine the glycaemic level in children and adolescents with T1DM	*n* = 82 all children and adolescents included consecutively; age range: 3–19 years, *n* = 39 (52.4%) males	Cross‐sectional study	A period of 6 months	Extraction of routine hospital health record data; sample of urine was collected and tested for ketones; Fasting blood glucose was measured
Noorani et al. (2016)	Tanzania	To identify the factors associated with glycaemic levels	*n* = 75; convenient sampling; mean age ± SD = 13.4 ± 3.9 years; almost equal sex distribution (50.7% males)	Cross‐sectional study	October 2010 to March 2011	Interviews
Shibeshi et al. (2016)	Ethiopia	To determine the prevalence of diabetic retinopathy	*n* = 86; consecutive; Mean age ± SD = 13.7 ± 1.8 years; males accounted for 45.3%	Cross‐sectional study	Not clear	Extraction of routine hospital health record data and interviews; Snellen distant vision test, Retinopathy was assessed using fundal photography
Shibeshi et al. (2022)	Ethiopia	To assess glycaemic level and identify its determinants	*n* = 116; consecutive; age ± SD = 13.1 ± 4.6 years (21 months to 18 years); *n* = 56 (48.3%) males	Cross‐sectional study	Not clear	Extraction of routine hospital health record data and interviews
Shimelash et al. (2023)	Ethiopia	To determine the incidence and predictors of mortality among children with DKA	*n* = 401; simple random sampling; mean ± SD age = 8.5 ± 2 years (including < 15 years); *n* = 215 (53.62%) were female	Retrospective follow‐up study	January 1, 2017, to December 31, 2021	Extraction of routine hospital health record data
Eshetu et al. (2024)	Ethiopia	To assess the risk and predictors of chronic microvascular complications of type 1 diabetes mellitus among children with diabetes	*n* = 132; convenient sampling; 52 (41.9%) are at pubertal age (10–14 years); 66 (53%) were males	Ambi directional cohort study	September 10, 2021 to January 30, 2023	Interviewing the primary caregivers for children under 12 years old and Interviewing children above 12 years old. Extraction of routine hospital health record data
Alemseged et al. (2024)	Ethiopia	To determine the prevalence and identify associated factors of lipodystrophy in children and adolescents with type 1 diabetes mellitus	*n* = 122; convenient sampling; the median age was 13 years, distribution led by adolescents at 65 (53.3%) and children at 57 (46.7%); 63 (51.6%) males, M: F ratio of 1.07:1	Cross‐sectional study design	May 1 to July 31, 2020	Interview visual inspection and palpation of the injection site by paediatric resident physicians.
AlehegnAwoke et al. (2024)	Ethiopia	To assess Prevalence and associated factors of psychiatric problems in children aged 6–18 years with type 1 diabetes mellitus	*n* = 206; convenient sampling technique; mean age of 12.8 years and SD of ±3.2; Males were higher (51.9%) than females (48.1%)	Cross‐sectional study design	February 1st to April 30th, 2022	interview using an interviewer‐administered structured questionnaire

Abbreviations: 95% CI, 95% Confidence Interval; AHR, adjusted hazard ratio; DKA, diabetic ketoacidosis; DM, diabetes mellitus; FBG, fasting blood glucose; HRQOL, health‐related quality‐of‐life; RBS, random blood sugar; RDA, Rwandan Diabetic Association; T1DM, type 1 diabetes mellitus.

Most studies used cross‐sectional designs (*n* = 22, 73.3%), while others employed retrospective follow‐up, observational surveys and cohort studies. Ten studies extracted data from routine hospital health records, while 11 combined hospital records with surveys; others incorporated face‐to‐face surveys, laboratory investigations, and anthropometric measurements (Table 1). A total of 6109 children and youths diagnosed with T1DM were enrolled in the included studies. The mean age of the participants was 10.6 years, and 3120 participants (51.1%) were males.

### 3.4 Review Question 1: Outcomes of T1DM

Fourteen studies in the review reported glycaemic levels (Abrahim et al. 2023; Alemseged et al. 2024; Eshetu et al. 2024; Habteyohans et al. 2023; Kayirangwa et al. 2018; Kidie et al. 2022; Lubwama 2022; Majaliwa et al. 2007; Msekandiana et al. 2020; Mukama et al. 2013; Ngwiri et al. 2015; Noorani et al. 2016; Shibeshi et al. 2022; Shibeshi et al. 2016); six reported on DKA (Assefa et al. 2020; Atkilt et al. 2017; Gebeyehu et al. 2022; Hadgu et al. 2019; Majaliwa et al. 2007; Msekandiana et al. 2020) and four on mortality associated with it (Gebeyehu et al. 2022; Meseret 2021; Musoma et al. 2020; Shimelash et al. 2023). Three studies reported on diabetic retinopathy (Majaliwa et al. 2007; Msanga et al. 2020; Shibeshi et al. 2016) and four on nephropathy (Lubwama 2022; Majaliwa et al. 2007; Msanga et al. 2020; Msekandiana et al. 2020), while three focused on HRQoL (Bekele et al. 2022; Gakuba 2016; Girma et al. 2021), and one reported psychiatric disorders (AlehegnAwoke et al. 2024) (Table 2).

**TABLE 2:** Outcome‐related characteristics of included studies.

Author/year	Outcome to be addressed	Outcome measured by	Reported outcomes	Factor associated with the outcome/s
Abrahim et al. (2023)	Glycaemic level (inability to achieve the agreed target level; associated factors)	Diagnosed as HbA1c; values ≥ 7.5% indicated above agreed target	Mean (SD) HbA1c 9.67% ± 2.28%; sample size affected = 121, indicating 76.6% did not achieve the agreed target level	Guardian or father as a primary caregiver [guardian (AOR = 4.45, 95% CI (1.03–19.16); *p* = 0.045), father (AOR = 6.02, 95% CI (1.58–22.96); *p* = 0.023)]; minimal involvement of caregiver in insulin injection (AOR = 5.39, 95% CI (1.86–15.60); *p* = 0.002); poor blood glucose monitoring adherence (AOR = 4.42; 95% CI (1.19–16.470); *p* = 0.026); facing problems at health facility (AOR = 4.42; 95% (1.24–9.52); *p* = 0.018); admission to hospital in past 6 months (AOR = 7.94; 95% CI (1.91–32.95); *p* = 0.004) were independently associated with the inability to achieve the agreed target level
AlehegnAwoke et al. (2024)	Assess the prevalence of Psychiatric disorders and associated factors	Psychiatric disorders among T1 DM patients were assessed using SDQ questions for mental health problems and the total difficulty Score, Eating disorders screening instruments (SCOFF)	The prevalence of psychiatric problems was 11.7%, eating disorders screening instruments (SCOFF) score was 0.30 (±0.6) and the Total SDQ score was 4.3 (±3.9)	Children living with only either of the parent (AOR = 8.39, 95% CI: 1.5–46), living with other relatives (AOR = 11.3, 95% CI: 1.97–64.7), more than 5 family size (AOR = 0.3, 95% CI: 0.1–1.2), fathers attended formal education (AOR = 0.3, 95% CI: 0.04–1.73), a patient having good glycaemic control (AOR = 0.2, 95% CI: 0.04–0.67) and those with a family history of diabetes mellitus (AOR = 5.2, 95% CI: 1.2–22.1) were significantly associated with a psychiatric problem
Alemseged et al. (2024)	Determine the prevalence of Lipodystrophy and identify associated risk factors; glycaemic level	Lipodystrophy was diagnosed as present or absent by visual inspection and palpation of the injection site. The presence of a noticeable or palpable lump at the injection site indicated that lipodystrophy was present. Accordingly, lipodystrophy was defined as having different grades based on morphology and pathogenesis; Optimal glycaemic control was when the recent HbA1c levels were less than or equal to 7.5%	Lipodystrophy was observed in 60 participants (49.2%, 95% CI: 40.4%–58.1%); HbA1c was conducted for 70 patients, and poor glycaemic control (HbA1c ≥ 7%) was found in 43 (61.4%) of them	Long duration of insulin injection (AOR = 3.6, 95% CI, 1.5–9.0, *p* = 0.005) and inappropriate rotation of the injection site (AOR = 9.0, 95% CI, 2.2–37.0, *p* = 0.002) were significantly associated with lipodystrophy
Assefa et al. (2020)	Incidence of diabetic ketoacidosis; predictors of diabetic ketoacidosis	Blood glucose measurement > 200 mg/dL or > 11 mmol/L and the presence of blood bicarbonate level < 15 mmol/L, and/or a pH < 7.30, and/or a DKA diagnosis mentioned in the medical records and/or ketone bodies in the urine	The incidence rate of DKA in the cohort was 2.27 cases per 100 children per month and the proportion of DKA was *n* = 207, 58.5%	Age < 5 years (AHR: 3.52, 95% CI (2.25, 5.49)), non‐adherence (AHR: 1.54, 95% CI (1.11, 2.14)), inappropriate insulin storage (AHR: 1.36, 95% CI (1.008, 1.85)), presence of upper respiratory tract infections during diabetic ketoacidosis diagnosis (AHR: 2.22, 95% CI (1.11, 4.45)) and preceding gastroenteritis (AHR: 2.18, 95% CI (1.07, 4.44)) were significant predictors DKA
Atkilt et al. (2017)	Proportion of DKA in children newly diagnosed with DM; factors associated with DKA in newly diagnosed children	Blood glucose level ≥ 250 mg/dL, ketonuria, and ketonemia in a patient being diagnosed with T1DM for the first time	Of children newly diagnosed with T1DM, the proportion with DKA = 142, 35.8%, 95% CI [31.6%, 40.8%]	Independent factors associated with DKA include: Age category 2 to 4.49 years (AOR = 3.14; 95% CI (1.21, 8.06)), 7 to 9.49 years (AOR = 3.44; 95% CI (1.39, 8.49)), and > 9.5 years (AOR = 4.02; 95% CI (1.68, 9.60)); parents' knowledge of the signs and symptoms of DKA (AOR = 0.51; 95% CI (0.27, 0.95)); signs and symptoms of DKA noted before the onset of DKA (AOR = 0.35; 95% CI (0.21, 0.59)) and infection before DKA onset (AOR = 3.45; 95% CI (1.97, 6.04))
Bacha et al. (2022)	Time required for resolution of DKA and associated factors; Hypoglycaemia; hypokalaemia; length of hospital stay	The time required for the resolution of DKA = calculated from the time at which management of DKA was started to the time management of DKA was discontinued; considered prolonged if > 12 h for a patient with mild DKA and > 36 h if moderate or severe DKA; Hypoglycaemia: RBS level less than 70 mg/dL; time between admission and discharge	The average time required for resolution of DKA was 48 ± 27.8 h; Hypoglycaemia *n* = 45, 23.7%; Hypokalaemia *n* = 8, 4.3%; Length of hospital stay was 7 days with a range of 2–20 days	Mental status on presentation (*p* = 0.001), shock on presentation (*p* < 0.01), and severity of DKA (*p* < 0.001) were found to have a significant association with the meantime for clearance of DKA. Age less than 1 year (AOR 9.53, CI 1.48–16.27, P,0.001) and age 1–5 years (AOR 4.54, CI 2.11–9.36, *p* < 0.001), Kussmaul's breathing (AOR = 3.47, ci 1.72–14.14, *p* < 0.001), shock on presentation (AOR = 4.93, CI 1.72–14.14, P 0.003), having severe DKA (AOR = 7.48, CI 3.04–18.42, *p* < 0.001) were significantly associated with the development of hypoglycaemia
Bekele et al. (2022)	The mean score of HRQoL and its associated factors among children and adolescents with T1D	HRQoL was assessed by measuring the mean score of HRQoL in children and adolescents using parents (the Proxy Report), as well as the children/adolescents Self‐Report assessment tool	The total mean ± SD score of HRQoL was 88.42 ± 10.82 (reported by the children and adolescents) and 82.17 ± 12.65 (reported by their primary caregivers)	According to self‐reports, age (β = −0.197; 95% CI (−1.131, −0.063); *p* = 0.028), mothers' having primary educational status (β = 0.242; 95% CI (2.859, 8.994); *p* < 0.001), mother having secondary educational status (β = 0.259; 95% CI (2.920, 8.831); fathers' having secondary educational status (β = 0.179 95% CI (0.080, 9.218)), *p* < 0.001), fathers' occupation (β = 0.170, *p* = 0.038), frequency of insulin administration (β = −0.132, *p* = 0.007), diabetes duration (β = −0.101, *p* = 0.050), and frequency of monitoring of blood glucose (β = 0.165, *p* = 0.006) were statistically significant predictors of HRQoL, explaining 21.6% of the variability of total HRQoL scores of children and adolescents (*R* ^2^ = 0.216, F (21,357) = 5.968, *p* < 0.001)
Eshetu et al. (2024)	Determining the time to development of complications from diagnosis of T1DM; glycaemic level	Urinal analysis for albuminemia, chemistry tests of serum creatinine, BUN, and cholesterol level using blood samples, peripheral neuropathy evaluation using the Douleur Neuropathique 4 questions, ophthalmologist evaluation using fundoscopic examination; Good glycaemic control: HbA1c level ≤ 7.5%	34 (27.4%) of the patients had at least one of the major chronic microvascular complications, overall incidence rate of chronic microvascular complications was 83 per 1000 population per year (95% CI: 59–116 per 1000 population per year), 103 (83%) had poor long‐term glycaemic control. The mean HgA1C level was 10.8 ± 2.5 mg/dL	Being male with IRR 1.71 (95% CI: 0.81–3.56), being at pubertal age IRR 1.91 (95% CI: 1.05–3.48), longer diabetes mellitus duration IRR 1.13 (95% CI: 1.07–1.28), and poor glycaemic control IRR 1.50 (95% CI: 0.46–4.97) were found to be at higher risk for chronic microvascular complication
Gakuba (2016)	Quality of life of children with diabetes and determining factors that lead to improved QOL	HRQoL was assessed by a short form of the Diabetes Quality of Life for Youth (DQOLY‐SF) questionnaire designed for measuring the quality of life in adolescents between 10 and 18 years	The mean ± SD = 20.08 ± 10.8; *n* = 92 (65.7%) = DQOLY‐SF score ≤ 21 (good quality of life) and *n* = 48 (34.3%) scored > 21 (poor QOL)	Age (*p*‐value 0.048), Hypoglycaemia episodes (*p*‐ 0.042), a province south or north (*p* < 0.005), and living with someone different from his/her parents (*p*‐ 0.008) were determinants for DQOLY‐SF score
Gebeyehu et al. (2022)	Proportion with DKA at new diagnosis; death	DKA was diagnosed by blood sugar levels at admission > 250 mg/dL + polyuria, polydipsia, polyphagia, and ketonemia/ketonuria. Severity graded as mild, moderate, and severe depending on clinical features	Proportion of DKA at new diagnosis was *n* = 173, 78.3%; death, *n* = 12, 6.9%	Factors independently associated with DKA: omission of insulin AOR 6.648; 95% CI (1.064–41.544); preceding infection: AOR 8.593; 95% CI (1.125–65.631); family history of DM: AOR 0.083; 95% CI (0.009–0.735). Cerebral oedema: AOR 0.007; 95% CI (0.00–0.114); infection: AOR 8.085; 95% CI (1.016–59.67); electrolyte imbalance: AOR 7.754; 95% CI (1.054–57.059); renal failure: AOR 0.018; 95% CI (0.002–0.203) were associated with mortality
Tsadik et al. (2018)	Magnitude of lipodystrophy and associated factors	Assessed by the presence of a palpable lump at the injection site	*n* = 103 (58.5%) = lipodystrophy; *n* = 100 (97.1%) lipohypertrophy; *n* = 3 (2.9%) lipoatrophy	The presence of insulin‐induced lipohypertrophy was significantly associated with the occurrence of nonoptimal glycaemic level (COR = 2.943, 95% CI: 1.303–6.649; *p* = 0 009)
Girma et al. (2021)	Quality of life and associated factors	Impaired HRQoL: mean score of more than 1 standard deviation (1SD) below the sample mean score of the self‐report in each subscale	Mean score = 78.8 ± 15.6 reported by the child and 61 ± 7.9 reported by the parents	Well‐controlled glycaemic level (β = 11.8, 95% CI: 8.7, 14.9), health education on diabetes (β = 5.92, 95% CI: 2.9, 8.9) and frequency of hospital admission (β = −2.6, 95% CI: −4.8, −0.42) were clinically predicting factors of health‐related quality of life
Habteyohans et al. (2023)	Inability to meet the agreed target glucose level; associated factors	Diagnosed by HbA1c > 7.5% and/or the average FBG level of either < 70 or > 145 mg/dL	Inability to meet the agreed target glucose level *n* = 166, 71.9% (95% CI: 66.0%–77.7%), Mean HgbA1c 10.4 ± 3.1 and mean FBG 220 ± 116	Age of the child (AOR = 0.19; 95% CI: 0.05–0.83; *p*: 0.027), education of the caregiver (AOR = 4.13; 95% CI: 1.82–9.46; *p*: 0.001), meal frequency less than three per day (AOR = 3.28; 95% CI: 1.25–8.62; *p*: 0.016), and consumption of forbidden foods (AOR = 3.17; 95% CI: 1.21–8.29; *p*: 0.019) were factors significantly associated with inability to meet the agreed target glucose level
Hadgu et al. (2019)	Proportion of diabetic ketoacidosis with newly diagnosed type 1 diabetes; associated factors	Poly‐symptoms, weight loss, vomiting, dehydration, Kussmaul breathing, lethargy or coma, and biochemically random blood sugar level > 11, glucosuria, and urine ketone > +1	Proportion of diabetic ketoacidosis with newly diagnosed T1DM *n* = 258, 78.7%	Age 5–10 years (AOR 0.24, 95% CI (0.06–0.95), P 0.042), 10.1–18 years (AOR 0.14, 95% CI (0.04–0.51), P 0.003), vomiting (AOR 2.73, 95% CI (1.29–5.80), P 0.009), excessive drinking (AOR 6.60, 95% CI (1.98–22.00), P 0.002), fatigue (AOR 2.22, 95% CI 1.17–4.22, P 0.015) and the presence of precipitating factor (AOR 7.81, 95% CI 2.97–20.50, *p* < 0.001) were associated with diabetic ketoacidosis with newly diagnosed T1DM
Kayirangwa et al. (2018)	The proportion of stunting; Inability to meet the agreed target glucose level	Stunting height for age is 2 standard deviations below the mean; underweight when weight for age is 2 standard deviations below the mean on WHO growth charts	Prevalence of stunting was 30.9%; the inability to meet the agreed target glucose level was 41.2%	Having an education below secondary school was associated with stunting. Females were less likely to be stunted than males (OR 0.3). Responders' perception of inadequate nutrition was associated with stunting
Kidie et al. (2021)	Frequency of DKA; determinant factors	Not clearly stated	The average frequency of DKA was 1.01 per individual (0–6); *n* = 188, 48.3% of the DM patients had at least one bout of DKA	Having an infection (AIRR = 1.41, 95% CI = 1.05–2.14), heart diseases (AIRR = 4.1, 95% CI = 1.17–14.68), treatment discontinuation (AIRR = 2.91, 95% CI = 2.02–4.22), low level of sodium (AIRR = 1.88, 95% CI = 1.22–2.89) and low dose of treatment at baseline (AIRR = 0.96, 95% CI = 0.94–0.97) were determinants factors
Kidie et al. (2022)	Proportion of inability to meet the agreed target glucose level; associated factors	Fasting blood glucose of < 70 mg/dL and > 130 mg/dL, HbA1c more or equal to 7%	The proportion of inability to meet the agreed target blood glucose level 39.3% (95% CI 34.6, 44.3)	Treatment discontinuation (AOR 2.42, 95% (CI 1.25, 4.69)), age (AOR 1.15, 95% (CI 1.03, 1.28)), and treatment dose (AOR 0.96, 95 CI (0.92, 0.99)) were significantly associated with the inability to meet the agreed target blood glucose level
Lubwama (2022)	Prevalence and associated factors of microalbuminuria and factors; Mean HbA1c	Diagnosed with elevated Albumin Creatinine Ratio (ACR) of 30 to 300 mg/g in males and 42 to 300 mg/g in females in 2 spot urine samples taken within 3 months	The prevalence of microalbuminuria = 13.7% (95% CI: 9.1%–20.2%). Only *n* = 120, 78% had HbA1c sample taken; mean (SD) HbA1c 11.2% ± 2.5%	Factors independently associated with microalbuminuria: duration of T1D less than 5 years (AOR 27.44, 95% CI (3.32, 226.77)); hospitalisation in the previous year (AOR 5.39, 95% CI (1.21–23.94)); hypertension (AOR 19.12, 95% CI (3.39, 107.83)); increased HbA1c (AOR 1.41, 95% CI (1.12–1.783))
Majaliwa et al. (2007)	Microalbuminuria; Retinopathy; glycaemic level; DKA at diagnosis of DM	Microalbuminuria = presence of protein, ketone, and sugar; Retinopathy = Fundus ophthalmoscopy; glycaemic level = laboratory measurement of HA1C	Microalbuminuria = 29, 29.3%; retinopathy: *n* = 22, 22.68%; inability to meet target glycaemic level: *n* = 98, 99%; DKA at diagnosis of DM: *n* = 75, 75%	
Meseret (2021)	Length of hospital stay, and in‐hospital mortality secondary to DKA and associated factors	Long hospital stay for more than seven days; DKA: blood glucose > 250 mg/dL and presence of ketonemia and ketonuria	mean length of hospital stay was 9.5 ± 6.2 days, 59.3% of had long hospital stay (> 7 days); In‐hospital mortality secondary to DKA was *n* = 4, 2.5%	Factors associated with long hospital stay were residence (AOR = 4.31; 95% CI = 1.25–14.80), family history of diabetes (AOR = 0.12; 95% CI = 0.02–0.64), glycemia at admission (AOR = 1.01; 95% CI = 1.00–1.02), insulin skipping (AOR = 0.08; 95% CI = 0.01–0.98), abdominal pain (AOR = 4.28; 95% CI = 1.11–15.52), time in which the patient get out of diabetic ketoacidosis (AOR = 6.39; 95% CI = 1.09–37.50)
Meseret et al. (2022)	Time to first optimal glycaemic level achievement; predictors	Optimal glycaemic achievement HbA1c < 7.5 and inability to achieve glycaemic target HbA1c > 7.5%; Time to the event‐ Time between initiation of treatment up to achieving first optimal glycaemic level	The median survival time to the first optimal glycaemic level was 8 months (95% CI: 6.9–8.9); the first optimal glycaemic achievement rate was 8.2 (95% CI: 7.2–9.2) per 100 person/month observation	Factors associated with time to first optimal glycaemic level were age > 10–14 years (AHR = 0.32; 95% CI = 0.19–0.55), increased weight (AHR = 0.96; 95% CI = 0.94–0.99), having primary caregiver (AHR = 2.09; 95% CI = 1.39–3.13), insulin dose (AHR = 1.05; 95% CI = 1.03–1.08), duration of diabetes ≥ 4 years (AHR = 0.64; 95% CI = 0.44–0.94), adherence to diabetic care (AHR = 9.72; 95% CI = 6.09–15.51), carbohydrate counting (AHR = 2.43; 95% CI = 1.12–5.26), and comorbidity (AHR = 0.72; 95% CI = 0.53–0.98)
Msanga et al. (2020)	Nephropathy, retinopathy, neuropathy, and associated risk factors	Diabetic nephropathy: presence of protein in the urine or eGFR less than 60 mL/min/1.73 m2; Diabetic retinopathy: exudates, haemorrhages, or new vessels at the disc or elsewhere in the fundus or fibrovascular membrane, reviewed by an ophthalmologist	*n* = 51, 32.9% had diabetic nephropathy, *n* = 16 had diabetic retinopathy (10.3%), and *n* = 21 had diabetic neuropathy (13.6%)	
Msekandiana et al. (2020)	Proportion of DKA and hypoglycaemia; microalbuminuria; Proportion of inability to meet the agreed target blood glucose level	Laboratory analysis of HBA1c; Microalbuminuria: albumin to creatinine ratio of ≤ 30–300 mg/g; Diabetic retinopathy: was assessed by slit lamp ophthalmoscopy	The proportion of DKA was 63.4%; severe hypoglycaemia (SH) *n* = 23, 56.10%: Proportion of inability to meet the agreed target blood glucose level *n* = 40, 97.56%; Microalbuminuria *n* = 2, 4.88%	Mode of insulin storage (*χ* ^2^ = 6.477, *p*‐ = 0.0039)
Mukama et al. (2013)	Impact of 6‐month diabetes management and Education programme on glycaemic level and acute complications in children and adolescents in Tanzania	The agreed target blood glucose level was assessed by HbA1c < 7.5% as good, HbA1c 7.5–10 as moderate, HbA1c 11%–12.5% as unable to meet the agreed target blood glucose level, and HbA1c > 12.5% as very poor level	*n* = 4 (5%) had HbA1c 7.5%, *n* = 22 (28%) had HbA1c between 7.5% and 10%, *n* = 9 (24%) had HbA1c between 11% and 12.5%, and *n* = 36 (44%) had HbA1c greater than 12.5%. *n* = 16 (10.3%) had diabetic retinopathy	
Musoma et al. (2020)	Proportion of mortality and associated factors; Average length of hospital stay	Mortality as confirmed by the physician recorded in the medical record of the child/adolescent	The proportion of mortality was *n* = 11, 6.9%; the median duration of hospital stay was 8 days	Mortality was associated with high serum creatinine (OR 5.8; 95% CI (1.6–21.2)), decreased urine output (OR 9.0; 95% CI (2.2–37.3)), and altered level of consciousness (OR 5.2; 95% CI (1.1–25.1))
Ngwiri et al. (2015)	Proportion of inability to meet the agreed target blood glucose level	HbA1c of 8% or less was a reasonable achievement and an inability to meet the agreed target blood glucose level if HbA1c was higher than 8%	The proportion of inability to meet the agreed target blood glucose level was 72%	Age above 12 years (AOR: 92.7, CI: 17.3–496.8, *p* < 0.0001) was significantly associated with the inability to meet the agreed target blood glucose level
Noorani et al. (2016)	Mean glycaemic level; factors associated with glycaemic level	The glycaemic level was assessed by mean HbA1c	Mean HbA1c was 11.1% ± 2.1%	Younger age, better adherence to blood glucose monitoring regimen, and having the mother as the primary caregiver were associated with better glycaemic levels (*R* ^2^ = 0.332, *p*‐ = 0.00)
Shibeshi et al. (2016)	Prevalence of diabetic retinopathy; the proportion of inability to meet the agreed target blood glucose level	Background or nonproliferative retinopathy: cottonwool spots, intra‐retinal haemorrhages, exudates, and microvascular abnormalities primarily in the macula and posterior retina	Background retinopathy *n* = 4, 4.7%; *n* = 45, 52.3% of the children did not meet the agreed target blood glucose level	
Shibeshi et al. (2022)	Glycaemic level; determinant factors	HbA1c value of < 7.5% (58 mmol/mol) = good glycaemic level while a value of ≥ 7.5% (58 mmol/mol) was an indicator of inability to meet the agreed target blood glucose level	Mean glycated haemoglobin (HbA1c) was 9.6% ± 2.4% (81 ± 3 mmol/mol). *n* = 97, 83.6% were not meet the agreed target blood glucose level [HbA1c ≥ 7.5% (58 mmol/mol)]	Presence of lipodystrophy changes at injection sites (AOR: 5.98, 95% CI (1.58, 22.73), *p*: 0.028) and being from a family that cannot afford insulin (AOR: 3.98, 95% CI: (1.63, 13.21), *p*: 0.028) were associated with inability to meet the agreed target blood glucose level
Shimelash et al. (2023)	Incidence and predictors of mortality among children in DKA	Death of the child at the specific time of follow‐up, as confirmed by the physician, is recorded in the medical record of the child/adolescent	The incidence of mortality of children with diabetic ketoacidosis was 10.6 per 1000 person‐days observed (95% CI: 7.8–14.4); proportion *n* = 40, 10%	Hypoglycaemia (AHR = 4.6; 95% CI: 2.13–10.1; *p*: 0.001), rural residence (AHR = 2.9; 95% CI = 1.01–8.11; *p*: 0.049), age younger than five (AHR = 4.4; 95% CI = 1.4–13.7; *p*: 0.011) or between five and 10 (AHR = 3.1; 95% CI = 1.1–8.8; *p*: 0.038), and female gender (AHR = 2.6; 95% CI = 1.1–5.8; *p*: 0.023) were significant predictors of mortality

Abbreviations: 95% CI, 95% Confidence Interval; AHR, adjusted hazard ratio; DKA, diabetic ketoacidosis; DM, diabetes mellitus; FBG, fasting blood glucose; HRQOL, health‐related quality‐of‐life; RBS, random blood sugar; RDA, Rwandan Diabetic Association; T1DM, type 1 diabetes mellitus.

#### 3.4.1 Glycaemic Level

A total of 14 studies involving 1781 children and youths with T1DM reported varying blood glucose levels, including the proportion and mean HbA1c values. Twelve studies reported the percentage of participants not achieving target glycaemic levels, and two studies provided only mean HbA1c values. The percentage of children and youths who did not achieve glycaemic target levels varied significantly, ranging from 39.3% to 99%. However, target values varied: six studies (50%) established a glycaemic threshold for HbA1c of 7.5% (Abrahim et al. 2023; Alemseged et al. 2024; Eshetu et al. 2024; Habteyohans et al. 2023; Mukama et al. 2013; Shibeshi et al. 2022); one study (10%) set the threshold at 8% (Ngwiri et al. 2015) and one study (5%) set it at 7% (Kidie et al. 2022). The remaining four studies (33%) did not specify a glycaemic threshold value (Kayirangwa et al. 2018; Majaliwa et al. 2007; Msekandiana et al. 2020; Shibeshi et al. 2016). The other two studies in the review only reported mean HbA1c levels and were not included in this threshold‐based classification.

The study that applied an HbA1c level of 7% as the threshold value found 39.3% of the participants were unable to meet this (Kidie et al. 2022). Of the six studies using 7.5% as the HbA1c glycaemic target, the percentage not achieving this ranged from 61.4% to 83.6% (Abrahim et al. 2023; Alemseged et al. 2024; Eshetu et al. 2024; Habteyohans et al. 2023; Mukama et al. 2013; Shibeshi et al. 2022). One study that applied an HbA1c threshold value of 8% reported 72% of the participants did not achieve this target (Ngwiri et al. 2015). Mean HbA1c values were reported from six studies, ranging from 9.6% to 11.2% (Eshetu et al. 2024; Habteyohans et al. 2023; Kidie et al. 2022; Lubwama 2022; Noorani et al. 2016; Shibeshi et al. 2022) (Tables 1 and 2).

#### 3.4.2 Diabetic Ketoacidosis

The percentage of participants who experienced DKA was reported in six studies, involving 1392 participants (Assefa et al. 2020; Atkilt et al. 2017; Gebeyehu et al. 2022; Hadgu et al. 2019; Majaliwa et al. 2007; Msekandiana et al. 2020). Between 35.8% and 78.7% of the participants presented with DKA at the initial diagnosis of T1DM (Hadgu et al. 2019) (Atkilt et al. 2017). Two other studies focused on the frequency of DKA (Kidie et al. 2021) and the average time required for its resolution (Bacha et al. 2022). However, these studies varied in their diagnostic criteria for DKA, which could have contributed to the differences in the reported proportions. Most studies also deviated from the international standard for the diagnosis of DKA (Glaser et al. 2022).

One study, which only adjusted the acidosis criterion, reducing the bicarbonate threshold value to < 15 mmol/L instead of the usual < 18 mmol/L, reported an incidence rate of 2.27 cases of DKA per 100 children per month. Of the 207 cases identified, 58.5% were confirmed as DKA (Assefa et al. 2020). Another study, which did not include an acidosis criterion and relied on detection of urine ketones at ≥ 1+ for diagnosis of DKA, reported that 78.7% of the participants were affected (Hadgu et al. 2019). Two studies, which applied a higher blood glucose threshold (≥ 250 mg/dL) and omitted acidosis markers, reported DKA rates of 35.8% and 78.3% (Atkilt et al. 2017; Gebeyehu et al. 2022).

Two studies did not provide diagnostic criteria for DKA based on blood glucose levels, acidosis, and ketosis, but reported that 75% and 63.4% of the participants experienced DKA (Majaliwa et al. 2007; Msekandiana et al. 2020). Two other studies, also not providing diagnostic thresholds for DKA, reported a frequency of DKA of 1.01 episodes per individual (Kidie et al. 2021) and the mean ± SD time to resolution of DKA was 48 ± 27.8 h from admission to discontinuation of treatment (Bacha et al. 2022) (Tables 1 and 2).

#### 3.4.3 Mortality

**FIGURE 2:**
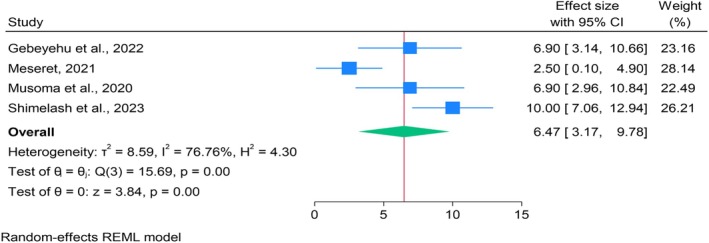
Forest plot of the pooled proportion of mortality among children with T1DM (*n* = 4).

Four studies involving 897 participants reported mortality rates among children and youths with DKA ranging from 2.5% to 10% (Gebeyehu et al. 2022; Meseret 2021; Musoma et al. 2020; Shimelash et al. 2023) (Table 2). Including all four studies, the pooled mortality estimate was 6.47% (95% CI: 3.17, 9.78%). Significant heterogeneity was observed (*Q* = 15.69, *p* < 0.001; *I* ^2^ = 96.71%), indicating substantial variability across studies (Figure 2). A Galbraith plot identified two studies as major contributors to this heterogeneity (Figure S1). Sensitivity analysis showed consistent estimates, ranging from 5.11% to 8.24% when each study was omitted (Figure S2).

Publication bias assessment using a funnel plot revealed asymmetry, prompting further testing (Figure S3). However, Egger's (*z* = 0.67, *p* = 0.503) and Begg's (*z* = −0.34, *p* = 1.000) tests showed no significant small‐study effects. A nonparametric trim‐and‐fill analysis imputed one study, slightly lowering the estimate to 5.21% (95% CI: 1.70%–8.72%), revealing no substantial change in the observed effect size. Overall, the findings were robust with minimal influence from publication bias.

#### 3.4.4 Health‐Related Quality of Life

Three studies assessed HRQoL in children and youths with T1DM (Bekele et al. 2022; Gakuba 2016; Girma et al. 2021). One study, employing the Diabetes Quality of Life for Youth (DQOLY‐SF) questionnaire, found that 65.7% of the participants had scores indicating good quality of life (Gakuba 2016) (Table 2). The other two studies used child and parent versions of the Paediatric Quality of Life inventory (PedsQL) to measure HRQoL. The first study reported mean HRQoL scores of 88.42 ± 10.82 from children and 82.17 ± 12.65 from parents (Bekele et al. 2022). In the second study, mean HRQoL scores were 78.8 ± 15.6 from children and 61 ± 7.9 from parents (Girma et al. 2021) (Table 2).

**FIGURE 3:**
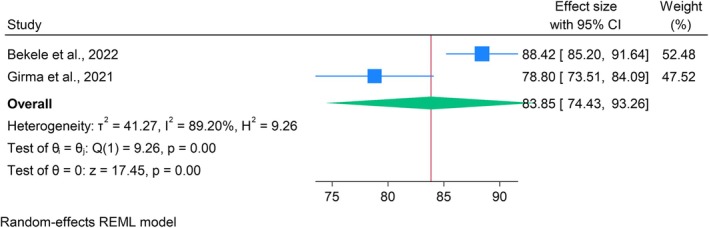
Forest plot of the pooled mean score of self‐reported HRQoL among children and youths with T1DM (*n* = 2).

**FIGURE 4:**
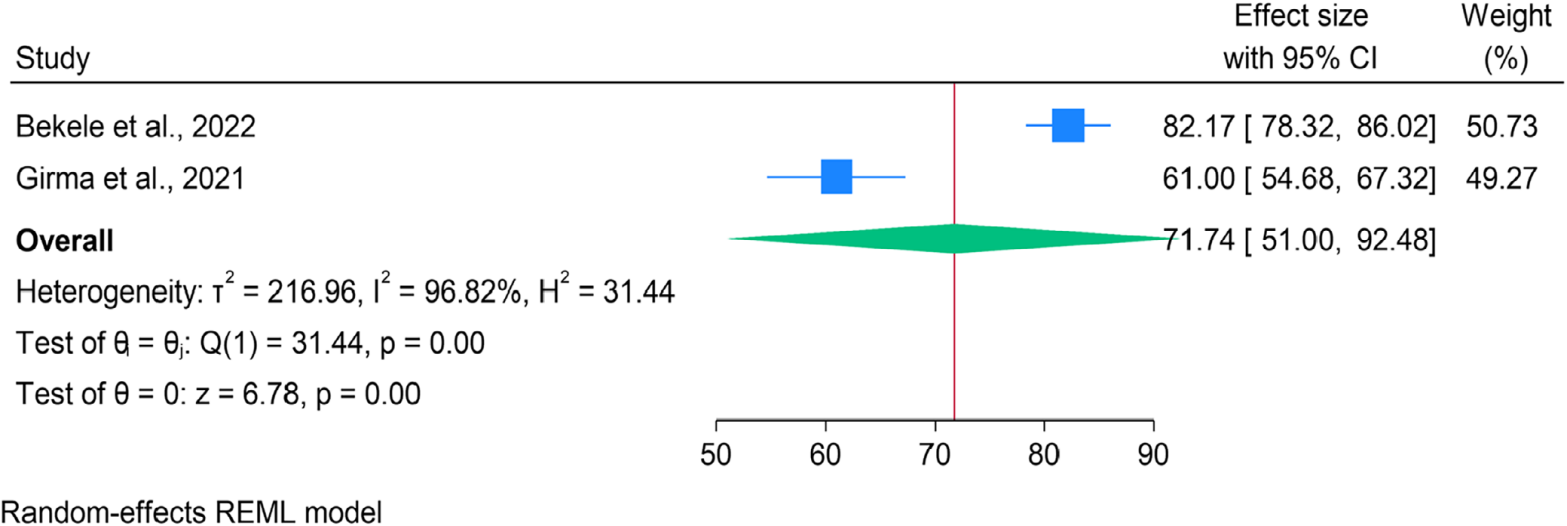
Forest plot of the pooled mean scores of parent‐reported HRQoL among children and youths with T1DM (*n* = 2).

Pooled analysis of two studies (Bekele et al. 2022; Girma et al. 2021) showed a mean self‐reported HRQoL of 83.85 (95% CI: 74.43–93.26) (Figure 3) and a parent‐reported HRQoL of 71.74 (95% CI: 51.00–92.48) (Figure 4). However, Q tests indicated significant heterogeneity in both self‐reported (*Q* = 9.62, *p* < 0.001) and parent‐reported (*Q* = 31.44, *p* < 0.001) HRQoL. Corresponding I^2^ statistics confirmed substantial heterogeneity, at 89.20% and 96.82%, respectively, suggesting considerable variation across the included studies (Figures 3 and 4).

#### 3.4.5 Other Adverse Outcomes

Beyond glycaemic levels, DKA, mortality, and HRQoL, other adverse outcomes were identified. Three studies reported rates of diabetic retinopathy, varying between 4.7% and 68% (Majaliwa et al. 2007; Msanga et al. 2020; Shibeshi et al. 2016) (Table 2). The applied diagnostic criteria and study designs used were suitable for pooling in meta‐analysis. The pooled proportion was 27.49% (95% CI: −11.99 to 66.97%) (Figure S4), though substantial heterogeneity was detected (*Q* = 151.32, *p* < 0.001; *I* ^2^ = 99.28%). Galbraith's analysis showed two studies contributed disproportionately to this heterogeneity (Figure S5), yet sensitivity analyses confirmed a persistently high estimate (Figure S6).

Similarly, nephropathy was reported in four studies, ranging from 88% to 29.3% (Lubwama 2022; Majaliwa et al. 2007; Msanga et al. 2020; Msekandiana et al. 2020) (Table 2), with design and similar diagnostic criteria allowing for meta‐analysis. The pooled estimate was 14.15% (95% CI: 4.47%–23.38%) (Figure S7), with high heterogeneity (*Q* = 19.53, *p* < 0.001; *I* ^2^ = 89.55%). A Galbraith plot indicated that two studies significantly influenced the observed variability (Figure S8), but sensitivity analysis validated that the prevalence estimates remained stable (Figure S9). One cohort study reported that 27.4% of the participants had at least one major microvascular complication, with an incidence rate of 83 per 1000 person‐years (95% CI: 59–116) (Eshetu et al. 2024).

Hospital admission due to T1DM‐related complications was also common and can itself be considered an adverse outcome (Tomic et al. 2022; Zhong et al. 2018). Three studies reported the length of stay due to T1DM‐related complications: one study reported a mean stay of 7 days (range: 2–20 days) (Bacha et al. 2022); another found 9.5 ± 6.2 days, with 59.3% staying over 7 days (Meseret 2021), and the third reported a median stay of 8 days (Mukama et al. 2013) (Table 2).

Two studies reported the prevalence of lipodystrophy: 58.5% in one study (Tsadik et al. 2018) and 49.2% in another (Alemseged et al. 2024). Other reported outcomes included psychiatric disorders, affecting 11.7% (AlehegnAwoke et al. 2024); stunting at 30.9% (Kayirangwa et al. 2018), and metabolic complications such as hypoglycaemia (23.7%) and hypokalaemia (4.3%) (Msekandiana et al. 2020) (Table 2).

### 3.5 Review Question 2. Factors Associated With Outcomes of T1DM

Several included studies identified factors significantly associated with not achieving target glycaemic levels in children and adolescents with T1DM. These factors included poor adherence to insulin therapy, inadequate glucose monitoring, and socioeconomic barriers, such as a family's inability to afford insulin. Other associated factors were older children, particularly those aged > 12 years, having a father as the primary caregiver, having a caregiver unable to read and write or having a lower level of education, minimal involvement of the caregiver in insulin administration, having families with a financial strain for insulin purchase, poor adherence to blood glucose monitoring, challenges at health facilities, hospital admissions in the past six months, meal frequency of fewer than three times a day, consumption of forbidden foods, insulin dosage, and the presence of lipodystrophy at injection sites. All these factors were independently linked to the achievement of glycaemic targets, demonstrated through multivariate regression analyses (Abrahim et al. 2023; Habteyohans et al. 2023; Kidie et al. 2022; Ngwiri et al. 2015; Shibeshi et al. 2022).

Studies reported several factors significantly associated with DKA among children and youths with newly diagnosed T1DM, including younger age, particularly in children under five years old, non‐adherence to insulin therapy, improper storage of insulin, low socioeconomic status, delayed diagnosis of T1DM, and insufficient diabetes education. Limited parental knowledge of DKA signs and symptoms, insulin omission, and a family history of diabetes also increased vulnerability. Infections such as gastroenteritis occurring prior to the occurrence of DKA also played an important role (Assefa et al. 2020; Atkilt et al. 2017; Gebeyehu et al. 2022; Hadgu et al. 2019).

Various factors were significantly associated with HRQoL in children and adolescents with T1DM. These included children whose mothers had no formal education, children with unemployed fathers, children receiving insulin injections three times per day, blood glucose monitoring done only during hospital follow‐ups, increased age of the child, a higher number of children in the family, female gender, frequent hospital admissions, and those with longer diabetes duration being linked to reduced HRQoL (Bekele et al. 2022; Girma et al. 2021). On the other hand, well‐controlled glycaemic levels and receiving diabetic education were positively linked with better HRQoL (Girma et al. 2021).

Several factors were identified as significantly associated with mortality in children and youths with T1DM. Clinical features such as cerebral oedema, infection, electrolyte imbalance, and renal failure conferred a higher risk of death (Gebeyehu et al. 2022). High serum creatinine, decreased urine output, and altered levels of consciousness were strongly linked to mortality (Musoma et al. 2020). Additionally, hypoglycaemia occurring due to mistreatment in children admitted with DKA, along with rural residence, younger age (particularly under 5 years), and female gender, were reported as contributing to higher mortality risk (Shimelash et al. 2023).

Male sex, age between 10 and 14 years, longer duration of diabetes, and not achieving glycaemic targets were linked with microvascular complications (Eshetu et al. 2024). Insulin‐induced lipohypertrophy was significantly associated with longer duration of insulin injection, inappropriate rotation of injection sites, and not meeting glycaemic targets (Alemseged et al. 2024; Tsadik et al. 2018).

Living with only one parent or other relatives, having larger family sizes (more than five members), and a family history of diabetes mellitus were linked with psychiatric disorders in children and youths with T1DM. Additionally, better parental education and achievement of glycaemic targets were linked to fewer psychiatric disorders (AlehegnAwoke et al. 2024).

## 4 Discussion

This review aimed to identify adverse outcomes associated with diabetes mellitus among children and youths in East Africa, and factors associated with these outcomes. Of 3797 publications searched across various databases for East African countries, 30 studies from only six nations were eligible for inclusion. Findings highlighted a number of critical adverse outcomes, including glycaemic level, DKA, mortality, microvascular complications, psychiatric disorders, and HRQoL among children and youths with T1DM in East Africa. High proportions of children and youths did not meet recommended blood glucose levels, and a notable proportion of this young population presented with DKA at their initial diagnosis of T1DM. Microvascular complications such as retinopathy and nephropathy were prevalent, and mortality and lower HRQoL associated with DKA were also critical concerns, underscoring the long‐term risks of T1DM.

The first key finding related to the high proportions of children and youths not meeting recommended glucose levels. Studies applied various threshold target values, which made it challenging to consider meta‐analysis. Nonetheless, even with higher than usual HbA1c thresholds, between 39.3% and 99% of the children and youths did not meet the recommended glucose levels. Additionally, mean HbA1c values across five studies ranged from 9.6% to 11.2%. This is important because blood glucose levels consistently above recommended target values can lead to complications including DKA, premature onset of microvascular complications, and long‐term health consequences (Bock et al. 2022).

These findings are consistent with data from Australia (Holmes‐Walker et al. 2023; James et al. 2022) and America (Demeterco‐Berggren et al. 2023), indicating that the problem is not unique to East Africa. These findings indicate widespread challenges in achieving optimal glycaemic levels in this population. This may be due to the nature of adolescence as a critical developmental stage, requiring tailored approaches that consider their unique physiological, psychological, and social needs. Additionally, in developing nations, systemic issues such as limited healthcare access and economic constraints may further exacerbate these challenges. This flags the urgent need for individualised, age‐appropriate strategies to address the unique challenges of this population. Healthcare professionals specialised in diabetes, particularly diabetes nurse educators, play a crucial role in addressing these challenges by providing personalised and holistic care.

To address barriers to the achievement of recommended glycaemic levels, it is crucial to identify and address the factors that contribute to these challenges. Findings addressing question two revealed several significant barriers, including personal, caregiver‐related, socioeconomic, and healthcare system‐related factors associated with glycaemic level. Among the most prominent barriers were poor adherence to insulin therapy and inadequate blood glucose monitoring. Socioeconomic barriers, such as families' inability to afford insulin, were also reported. Caregivers' characteristics were negatively linked with glycaemic achievement, such as where the primary caregiver was the father, or unable to read and write, was poorly educated, or had minimal involvement in insulin administration. Other factors associated with poor outcomes included older age of the child (> 12 years), recent hospital admissions, and nutritional factors like fewer than three meals a day and restricted food choices. Health system challenges were also linked with poor outcomes, including long wait times, lack of specialised care, and other problems encountered at the hospital while seeking health services (Abrahim et al. 2023; Habteyohans et al. 2023; Kidie et al. 2022; Ngwiri et al. 2015; Shibeshi et al. 2022).

These barriers highlight the wide range of challenges that need to be addressed to enhance outcomes for children and youths with T1DM, related to individual behaviour, caregiver support, socioeconomic assistance, and improvements in health service delivery. It is important that healthcare professionals who specialise in diabetes, such as diabetic nurse educators, are available to develop effective strategies to support patients and their families in achieving better health outcomes.

Another finding of the review was the high proportion of this population who experienced DKA at the initial diagnosis of T1DM, with six studies reporting rates ranging from 35.8% to 78.7% (Table 2). These figures are predominantly higher than the findings of studies from America (Praveen et al. 2021), European countries (Nagl et al. 2022) and Australia (Ampt et al. 2019). This disparity likely arises from variations in access to healthcare facilities and diagnostic resources, education and awareness about the disease, and socioeconomic differences.

The review further identified factors that increased vulnerability and influenced the onset and management of DKA in these patients with T1DM, including a family history of diabetes, a younger age of the child, and a delayed diagnosis of T1DM, low socioeconomic status, insufficient diabetes education, and limited parental knowledge of DKA resulting in non‐adherence to insulin therapy such as improper storage of insulin and insulin omission (Assefa et al. 2020; Atkilt et al. 2017; Gebeyehu et al. 2022; Hadgu et al. 2019). Infections occurring prior to the occurrence of DKA also played an important role (Atkilt et al. 2017). Altogether, this emphasised the role of clinical, behavioural, and social factors in the occurrence of DKA. Importantly, these factors, especially infections such as pneumonia, are often confused with DKA and misdiagnosed in young children, which can delay intervention, emphasising the need for a high index of suspicion and diagnostic accuracy for DKA.

The implications of these findings are significant for practice, particularly in resource‐limited settings. Strategies such as managing infections effectively, early diagnosis of T1DM, improving education not just on DKA warning signs but daily diabetes management to improve adherence to insulin are all important. Addressing these multiple and complex issues will require comprehensive interventions to improve healthcare access, enhance education, mitigate economic disparities, and foster culturally sensitive healthcare practices. In resource‐limited settings, targeted education and early intervention strategies, such as community‐based programmes and training local healthcare providers, are key to improving DKA management and outcomes. Such efforts are essential to support glycaemic management in line with international target values, reduce the incidence of DKA, and improve outcomes for individuals with T1DM in East Africa.

Review findings also identified concerning mortality rates among children and youths with diabetes in East Africa, with four studies reporting rates ranging from 2.5% to 10%, with a pooled proportion of 6.47% (95% CI: 3.17, 9.78%). Largely attributed to the high proportions diagnosed with DKA, these high rates of death may also result from delayed diagnosis, inadequate access to healthcare services, insufficient diabetes management programmes, and lack of patient and family education on diabetes self‐care (Beck et al. 2017). Therefore, improved access to healthcare services, comprehensive diabetes management programmes, and enhanced education on diabetes self‐care are crucial to save lives (Nam Hoon 2019).

Finally, significant proportions of children and youths with T1DM were reported with microvascular complications. Diabetic retinopathy affected a pooled proportion of 27.49%, while nephropathy affected 14.15%. These complications, associated with longer duration of T1DM and inability to achieve glycaemic targets, reinforce the need for early interventions and sustained management. Not just attainment but sustained maintenance of recommended blood glucose values, enabled by routine screening and timely intervention, is essential to prevent and delay progression of these complications (Bjornstad et al. 2022).

### 4.1 Strengths and Limitations of the Review

This review offers a comprehensive assessment of key outcomes related to T1DM reported for children and youths in East Africa which, where available, provide valuable insights into diabetes management and its impacts in some of these settings. Its strengths include a detailed examination of critical complications associated with T1DM, including glycaemic levels, DKA, diabetic retinopathy, nephropathy, and mortality, across diverse geographical and socioeconomic settings, particularly in resource‐limited areas. However, the review has limitations.

First, studies only originated from six of the potential 22 nations, states, and departments of East Africa. As it is impossible to determine how representative this work may be, further work is required across other areas of this region, and future studies should take note of the limitations revealed by this review.

The heterogeneity of outcome measurements across studies for glycaemic level and DKA may have impacted the comparability of findings. Deviation of outcome diagnostic criteria from internationally agreed values, heterogeneity in outcome diagnosis thresholds between studies, limitations in identifying and managing confounding factors, and overlooking important variables were notable limitations that precluded the conduct of meta‐analyses for glycaemic levels and DKA, hindering meaningful data aggregation and comparison.

The review highlighted the gap in research regarding T2DM in children and youths, an enormously important deficit considering its fast‐growing prevalence and impact in other parts of the world. Although global evidence is growing on the role of molecular markers like Sirtuin 1, no studies examined this, which precluded consideration of any potential contribution.

Despite these limitations, the review underscores the need for future research to address the identified gaps, to provide data to support improvement in the quality of diabetes care, particularly in resource‐limited settings, and to expand the focus to include T2DM for a more comprehensive understanding of diabetes‐related complications.

## 5 Conclusion

This review highlights the urgent need for context‐specific and personalised care strategies to effectively address the challenges of diabetes management in children and youths, particularly in resource‐limited regions such as those that occur in East Africa.

From a clinical practice perspective, the role of specialised professionals, particularly diabetes nurse educators, is critical. Their expertise in early diagnosis, glycaemic monitoring, and development and delivery of comprehensive, locally tailored patient and family education enables the delivery of holistic care that supports both physical and psychosocial well‐being. Their continued engagement is central to improving disease management and mitigating adverse outcomes in this vulnerable population. In terms of policy implications, efforts must prioritise the strengthening of healthcare infrastructure including the diabetes workforce, the expansion of health insurance schemes, and the implementation of public health education initiatives. Policymakers should also focus on community‐based education and social support systems to better manage the growing burden of diabetes in this population.

Looking ahead, researchers should address the gaps identified in this review through longitudinal, interventional, mixed‐method, and qualitative inquiries to deepen understanding of disease trajectories and intervention effectiveness, including for new developments such as biomarkers. Given the growing rates in other regions, there is an urgent need for studies on type 2 diabetes in children and youths, which the review revealed as unexplored in this region.

## Author Contributions

Chalie Marew Tiruneh, Marilyn Cruickshank, Muhammad Chutiyami, Lin Perry made substantial contributions to conception and design, or acquisition of data, or analysis and interpretation of data. Involved in drafting the manuscript or revising it critically for important intellectual content. All authors have given final approval of the version to be published. Each author has participated sufficiently in the work to take place for appropriate portions of the content. Agreed to be accountable for all aspects of the work in ensuring that questions related to the accuracy or integrity of any part of the work are appropriately investigated and resolved.

## Ethics Statement

The authors have nothing to report.

## Consent

The authors have nothing to report.

## Conflicts of Interest

The authors declare no conflicts of interest.

## Supporting information


**Data S1:** jan70124‐sup‐0001‐DataS1.docx.

**Data S2:** jan70124‐sup‐0002‐DataS2.docx.

## Data Availability

The data that supports the findings of this study are available in the Supporting Information of this article.
