# Supplementary material for: Adverse Outcomes and Associated Factors Among Children and Youths With Diabetes Mellitus in East Africa: A Systematic Review and Meta‐Analysis
Source: J Adv Nurs. 2025 Aug 4;82(4):2659–84. doi: 10.1111/jan.70124 (PMC12994644; doi:10.1111/jan.70124)
Supplement: Supplementary file 1 — Data S1: jan70124‐sup‐0001‐DataS1.docx. [file JAN-82-2659-s001.docx]

**Supplementary File 1: Tables**

Supplementary Table 1. Literature search terms.

| Database | MEDLINE and Embase | CINAH | Scopus | Web of Science databases |
| --- | --- | --- | --- | --- |
| Line 1 | Child, Preschool/ or Child/ or child*.mp. or Adolescent/ or adolescent.mp. or p? ediatrics.mp. or Pediatrics/ or teenager. mp. or Adolescent/ | "child*" OR (MH "Child") OR "adolescents" OR "teens" OR (MH "Adolescence") OR "p#ediatrics" OR (MH "Pediatrics") | ((CHILD*) OR (ALL (ADOLESCENT*)) OR (ALL (pediatrics)) OR (ALL (PEDIATRICS)) OR (ALL (TEENAGER)) OR ( ALL ( school age ) ) ) | ((((TS=(child*)) OR TS=(pediatrics)) OR TS=(paediatrics)) OR TS=(adolescen*)) OR TS=(teenager) and Preprint Citation Index (Exclude – Database) |
| AND | | | | |
| Line 2 | diabetes.mp. or Diabetes Mellitus, Type 2/ or Diabetes Mellitus/ or Diabetes Mellitus, Type 1/ or insulin-dependent diabetes mellitus.mp. or Diabetes Mellitus, Type 1/ or Non-insulin-dependent diabetes mellitus.mp. or Diabetes Mellitus, Type 2/ or Type 1 diabetes mellitus.mp. or Diabetes Mellitus, Type 1/ or Type 2 diabetes mellitus.mp. or Diabetes Mellitus, Type 2/ or Type I diabetes mellitus.mp. or Type II diabetes mellitus.mp. or Diabetes mellitus, type I.mp. or Diabetes mellitus, type II.mp. | MH "Diabetes Mellitus, Type 2") OR (MH "Diabetes Mellitus, Type 1") OR "diabetes"OR (MH "Diabetes Mellitus") OR "diabetes mellitus" OR (MH "Diabetes Mellitus") OR "diabetes mellitus"  OR "type 1 diabetes mellitus" OR "Type 2 diabetes or type 2 diabetes mellitus or t2dm" OR "Type 2 diabetes mellitus" OR "Type I diabetes mellitus" OR "Type II diabetes mellitus" OR "non-insulin-dependent diabetes mellitus" OR "Insulin-dependent diabetes mellitus" | ( ( TITLE-ABS-KEY ( diabetes ) ) OR ( TITLE-ABS-KEY ( "diabetes mellitus" ) ) OR ( TITLE-ABS-KEY ( "type 1 diabetes mellitus" ) ) OR ( TITLE-ABS-KEY ( "type I diabetes mellitus" ) ) OR ( TITLE-ABS-KEY ( "type 2 diabetes mellitus" ) ) OR ( TITLE-ABS-KEY ( "type ii diabetes mellitus" ) ) OR ( TITLE-ABS-KEY ( "insulin-dependent diabetes mellitus" ) ) OR ( TITLE-ABS-KEY ( "non-insulin-dependent diabetes mellitus" ) ) OR ( TITLE-ABS-KEY ( "t1dm" ) ) OR ( TITLE-ABS-KEY ( "t2dm" ) ) ) | (((((((TS= (diabetes mellitus)) OR TS=(diabet*)) OR TS=(type 1 diabetes)) OR TS=(type 2 diabetes)) OR TS=(Insulin dependent diabetes mellitus)) OR TS=(non-insulin dependent diabetes mellitus)) OR TS=(type I diabetes mellitus)) OR TS=(Type II diabetes mellitus) and Preprint Citation Index (Exclude – Database) |
| AND | | | | |
| Line 3 | Burundi/ or Burundi.mp. or Comoros.mp. or Comoros/ or Djibouti.mp. or Djibouti/ or Eritrea.mp. or Eritrea/ or Ethiopia.mp. or Ethiopia/ or Kenya.mp. or Kenya/ or Madagascar.mp. or Madagascar/ or Rwanda.mp. or Rwanda/ or Seychelles.mp. or Seychelles/ or Somalia.mp. or Somalia/ or Somaliland.mp. or Malawi/ or Mauritius/ or Tanzania.mp. or Tanzania/ or Uganda.mp. or Uganda/ or South Sudan/ or Mozambique.mp. or Mozambique/ or Zambia.mp. or Zambia/ or Zimbabwe.mp. or Zimbabwe/ or British Indian Ocean Territory.mp. or Reunion.mp. or Reunion/ or Mayotte.mp. or French Southern Territories.mp. | "Burundi" OR "Comoros"  OR "Djibouti" OR "Eritrea"  OR (MH "Ethiopia") OR "Ethiopia" OR (MH "Kenya")  OR (MH "Madagascar") OR "Madagascar" OR "Rwanda" OR "Malawi"  OR "Mauritius" OR "Seychelles" OR "Somalia"  OR "Somaliland" OR "South Sudan" OR "Tanzania"  OR "Uganda" OR “Mozambique” OR "Zambia" OR "Zimbabwe” OR "Indian Ocean Territory" OR "Reunion" OR "Mayotte" OR "French Southern Territories" | ( ( TITLE-ABS-KEY ( uganda ) OR TITLE-ABS-KEY ( tanzania ) OR TITLE-ABS-KEY ( "south sudan" ) OR TITLE-ABS-KEY ( somaliland ) OR TITLE-ABS-KEY ( somalia ) OR TITLE-ABS-KEY ( seychelles ) OR TITLE-ABS-KEY ( mauritius ) OR TITLE-ABS-KEY ( malawi ) OR TITLE-ABS-KEY ( rwanda ) OR TITLE-ABS-KEY ( madagascar ) OR TITLE-ABS-KEY ( kenya ) OR TITLE-ABS-KEY ( ethiopia ) OR TITLE-ABS-KEY ( eritrea ) OR TITLE-ABS-KEY ( djibouti ) OR TITLE-ABS-KEY ( burundi ) OR ( TITLE-ABS-KEY ( mozambique ) ) OR ( TITLE-ABS-KEY ( zambia ) ) OR ( TITLE-ABS-KEY ( zimbabwe ) ) OR ( TITLE-ABS-KEY ( mayotte ) ) OR ( TITLE-ABS-KEY ( reunion ) ) OR ( TITLE-ABS-KEY ( "French Southern Territories" ) ) OR ( TITLE-ABS-KEY ( "British Indian Ocean Territory" ) ) ) | (((((((((((((((((((TS=(Burundi)) OR TS=(Djibouti)) OR TS=(eritrea)) OR TS=(Ethiopia)) OR TS=(Kenya)) OR TS=(madagascar)) OR TS=(malawi)) OR TS=(mauritius)) OR TS=(Rwanda)) OR TS=(Seychelles)) OR TS=(South Sudan)) OR TS=(Somalia)) OR TS=(somaliland)) OR TS=(Tanzania)) OR TS=(Uganda)) OR TS=(Mozambique)) OR TS=(Zambia)) OR TS=(Zimbabwe)) OR TS=(Mayotte)) OR TS=(French Southern Territories)) OR TS=(Reunion)) OR TS=(British Indian Ocean Territory)) and Preprint Citation Index (Exclude – Database) |

Supplementary Table 2: Data items of the review and their definition

| Data item | Definition | Reference |
| --- | --- | --- |
| Glycemic level | The management of blood glucose within the recommended target range to prevent acute and chronic complications of diabetes. It is operationally defined based on HbA1c levels, with optimal glycemic control classified as HbA1c <7% (<53 mmol/mol), as per the ISPAD Clinical Practice Consensus Guidelines 2022 for children and adolescents | (de Bock et al., 2022) |
| Diabetic ketoacidosis: | The presence of hyperglycemia (blood glucose >11 mmol/L or ≈200 mg/dL), acidosis (venous pH <7.3 or serum bicarbonate <18 mmol/L), and ketosis (blood β-hydroxybutyrate ≥3 mmol/L or moderate to large ketonuria). Clinical manifestations may include polyuria, polydipsia, vomiting, dehydration, Kussmaul breathing, lethargy, or coma | (Glaser et al., 2022) |
| Health-related Quality of Life (H-RQoL) | The physical, school, emotional, psychosocial, and social domains of health can be influenced by a person’s experiences, beliefs, expectations, and perceptions | (Zaror et al., 2019). |
| Diabetic nephropathy | A complication of diabetes that affects the kidneys, which is characterized by persistent albuminuria and decreased glomerular filtration rate | (Bjornstad et al., 2022) |
| Diabetic retinopathy | A complication of diabetes that affects the eyes, resulting in exudates, haemorrhages or new vessels in the eye, which can cause vision loss and blindness, diagnosed by expert clinical assessment | (Bjornstad et al., 2022) |
| Length of hospital stay | the time between hospital admission and discharge. |  |

Supplementary Table 3: Quality appraisal

A: Quality appraisal status of cross-sectional design studies(Moola S, 2020a)

| Studies | Criteria scores for four responses: 1. Yes, 0. No 0. Unclear 0. Not applicable (NA) | | | | | | | | Total of max. 8 |
| --- | --- | --- | --- | --- | --- | --- | --- | --- | --- |
|  | 1 | 2 | 3 | 4 | 5 | 6 | 7 | 8 |  |
| (Bacha et al., 2022) | Yes | Yes | Yes | Yes | Unclear | No | Yes | Yes | 6 |
| (Gebeyehu et al., 2022) | Yes | Yes | Yes | Yes | No | No | Yes | Yes | 6 |
| (Girma et al., 2021) | Yes | Yes | Unclear | Unclear | Unclear | No | Yes | Yes | 4 |
| (Ngwiri et al., 2015) | Yes | Yes | Yes | Yes | Unclear | No | Yes | Yes | 6 |
| (Noorani et al., 2016) | Yes | Yes | Yes | Yes | Unclear | No | Unclear | Yes | 5 |
| (Abrahim et al., 2023) | Yes | Yes | Yes | Yes | Unclear | Yes | Yes | Yes | 7 |
| (Shibeshi et al., 2016) | Yes | Yes | Yes | Yes | Unclear | Unclear | Yes | Yes | 6 |
| (Habteyohans et al., 2023) | Yes | Yes | Yes | Yes | Unclear | Yes | Yes | Yes | 7 |
| (Lubwama, 2022) | Yes | Yes | Yes | Yes | Unclear | Unclear | Yes | Yes | 6 |
| (Gebremeskel Tsadik et al., 2018) | Yes | Yes | Yes | Yes | Unclear | Yes | Yes | Yes | 7 |
| (Msekandiana et al., 2020) | Yes | Yes | Yes | Yes | Unclear | Yes | Unclear | Yes | 6 |
| (Hadgu et al., 2019) | Yes | Yes | Yes | Yes | Unclear | No | Yes | Yes | 6 |
| (Atkilt et al., 2017) | Yes | Yes | Yes | Yes | Unclear | No | Yes | Yes | 7 |
| (Kidie et al., 2021) | Yes | Yes | Yes | Yes | Unclear | No | Unclear | Yes | 5 |
| (Msanga et al., 2020) | Yes | Yes | Yes | Yes | Unclear | Yes | Yes | Yes | 7 |
| (GAKUBA, 2016) | Yes | Yes | Yes | Unclear | Unclear | Unclear | Yes | Yes | 5 |
| (Kayirangwa et al., 2018) | Yes | Yes | Yes | Unclear | Yes | Yes | Yes | Unclear | 6 |
| (Kidie et al., 2022) | Yes | Yes | Yes | Yes | Unclear | Yes | Yes | Yes | 7 |
| (Shibeshi et al., 2022) | Yes | Yes | Yes | Yes | Unclear | Yes | Yes | Yes | 7 |
| (Majaliwa et al., 2007) | Yes | Yes | Yes | Yes | Unclear | Unclear | Yes | Yes | 6 |
| (Bekele et al., 2022) | Yes | Yes | Yes | Unclear | Unclear | Unclear | Yes | Yes | 5 |
| (Musoma et al., 2020) | Yes | Yes | Yes | Yes | Unclear | Unclear | Yes | Yes | 6 |
| (Meseret, 2021) | Yes | Yes | Yes | Yes | Unclear | Unclear | Yes | Yes | 6 |
| (Mukama et al., 2013) | Yes | Yes | Yes | Unclear | Unclear | No | Yes | Yes | 5 |
| (Alemseged et al., 2024) | Yes | Yes | Yes | Yes | Unclear | Yes | Yes | Yes | 7 |
| (AlehegnAwoke et al., 2024) | No | Yes | Yes | Yes | Unclear | Unclear | Yes | Yes | 6 |
| Tool items, (<https://jbi.global/sites/default/files/2021-10/Checklist_for_Analytical_Cross_Sectional_Studies.docx> )   1. Were the criteria for inclusion in the sample clearly defined? 2. Were the study subjects and the setting described in detail? 3. Was the exposure measured validly and reliably? 4. Were objective, standard criteria used for measurement of the condition? 5. Were confounding factors identified? 6. Were strategies to deal with confounding factors stated? 7. Were the outcomes measured validly and reliably? 8. Was appropriate statistical analysis used? | | | | | | | | | |

B. Quality appraisal status of cohort studies (Moola S, 2020b)

|  | Studies | Criteria scores for four responses: 1. Yes, 0. No 0. Unclear 0. Not applicable (NA) | | | | | | | | | |  |  |
| --- | --- | --- | --- | --- | --- | --- | --- | --- | --- | --- | --- | --- | --- |
|  |  | 1 | 2 | 3 | 4 | 5 | 6 | 7 | 8 | 9 | 10 | 11 | Total of max. 11 |
|  | (Assefa et al., 2020) | Na | Na | Yes | Unclear | Unclear | Yes | Yes | Yes | Yes | Yes | Yes | 7 |
|  | (Shimelash et al., 2023) | Yes | Yes | Yes | Unclear | Yes | Yes | Yes | Yes | Yes | Yes | Yes | 10 |
|  | (Meseret et al., 2022) | Yes | Yes | Yes | Unclear | Yes | Yes | Yes | Yes | Yes | Yes | Yes | 10 |
|  | (Eshetu et al., 2024) | Yes | Yes | Yes | Unclear | Yes | Yes | Yes | Yes | Yes | NA | Yes | 9 |
| Tool items, (<https://jbi.global/sites/default/files/2021-10/Checklist_for_Cohort_Studies.docx> )   1. Were the two groups similar and recruited from the same population? 2. Were the exposures measured similarly to assign people to both exposed and unexposed groups? 3. Was the exposure measured validly and reliably? 4. Were confounding factors identified? 5. Were strategies to deal with confounding factors stated? 6. Were the groups/participants free of the outcome at the start of the study (or at the moment of exposure)? 7. Were the outcomes measured validly and reliably? 8. Was the follow-up time reported sufficient to be long enough for outcomes to occur? 9. Was follow-up complete, and if not, were the reasons for loss to follow-up described and explored? 10. Were strategies to address incomplete follow-up utilized? 11. Was appropriate statistical analysis used? | | | | | | | | | | | | | |
