# Supplementary material for: Adverse Outcomes and Associated Factors Among Children and Youths With Diabetes Mellitus in East Africa: A Systematic Review and Meta‐Analysis
Source: J Adv Nurs. 2025 Aug 4;82(4):2659–84. doi: 10.1111/jan.70124 (PMC12994644; doi:10.1111/jan.70124)
Supplement: Supplementary file 2 — Data S2: jan70124‐sup‐0002‐DataS2.docx. [file JAN-82-2659-s002.docx]

*Supplementary File 2: Figures*


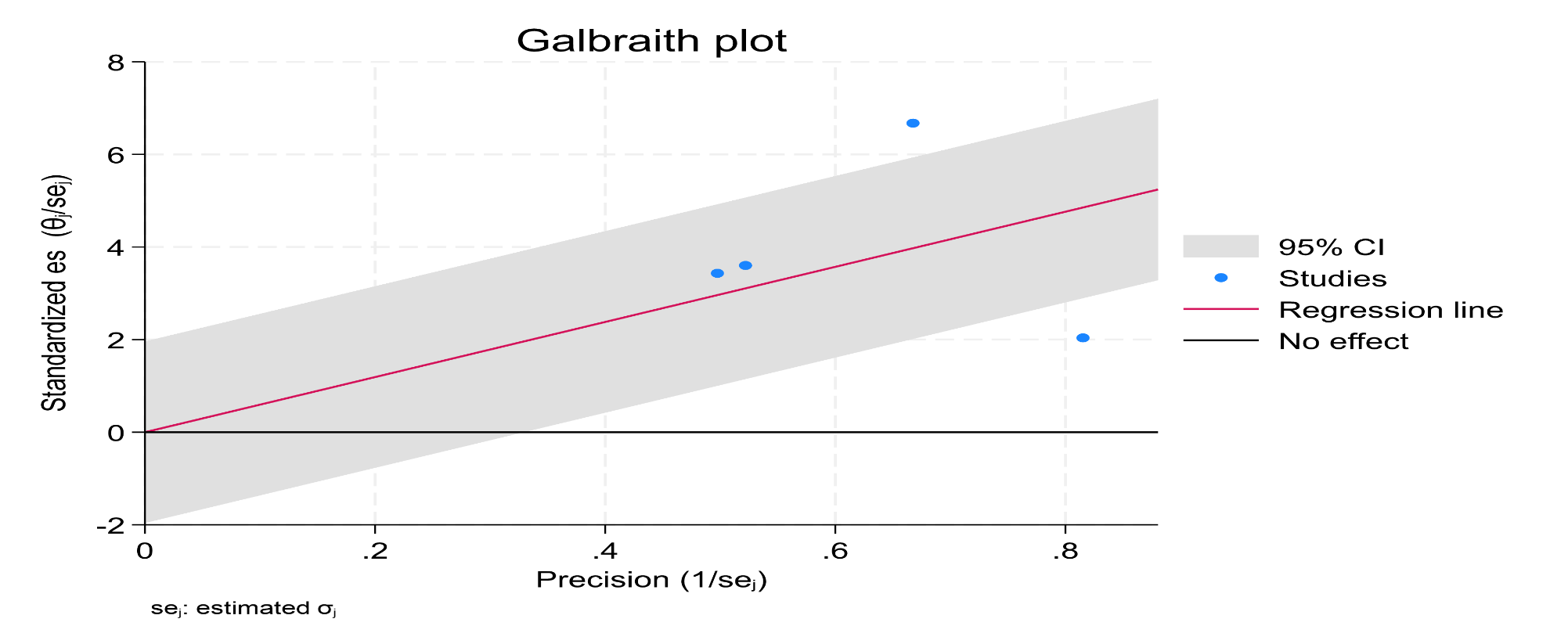


Supplementary Figure 1. Galbraith plot to investigate the source of heterogeneity of proportion of mortality.


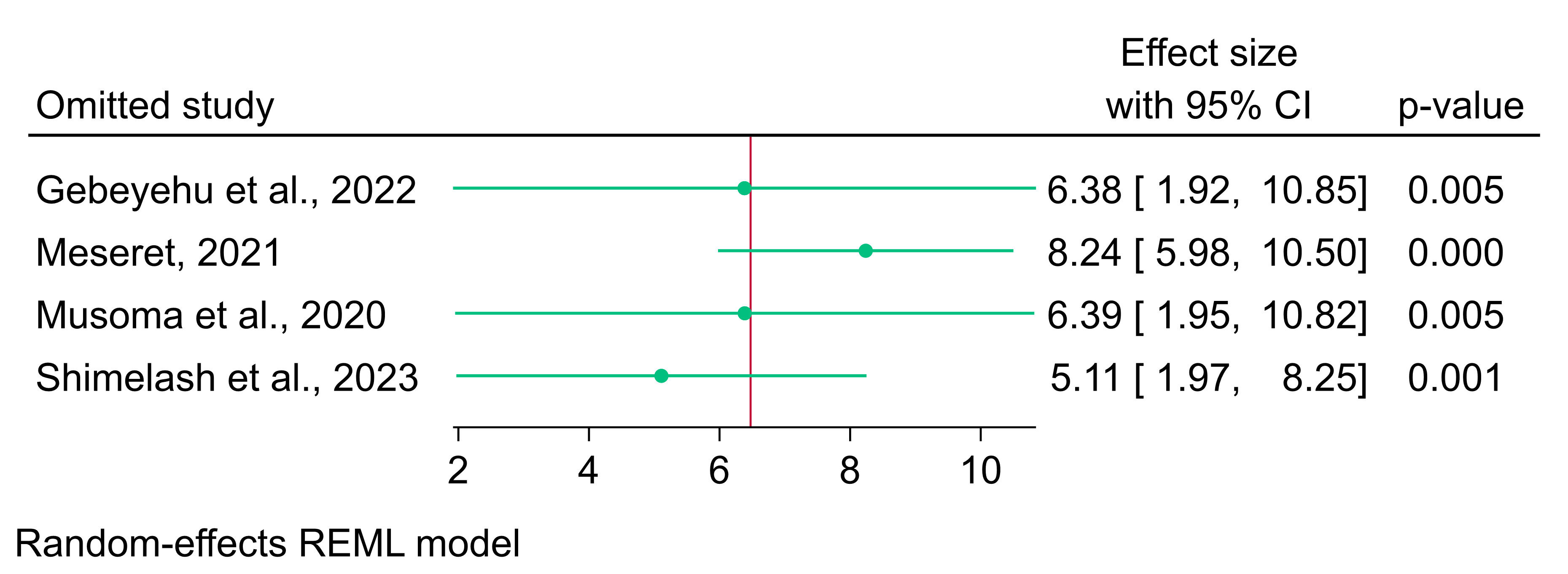


Supplementary Figure 2. A leave-one-out meta-analysis to investigate the source of heterogeneity of proportion of mortality.

*
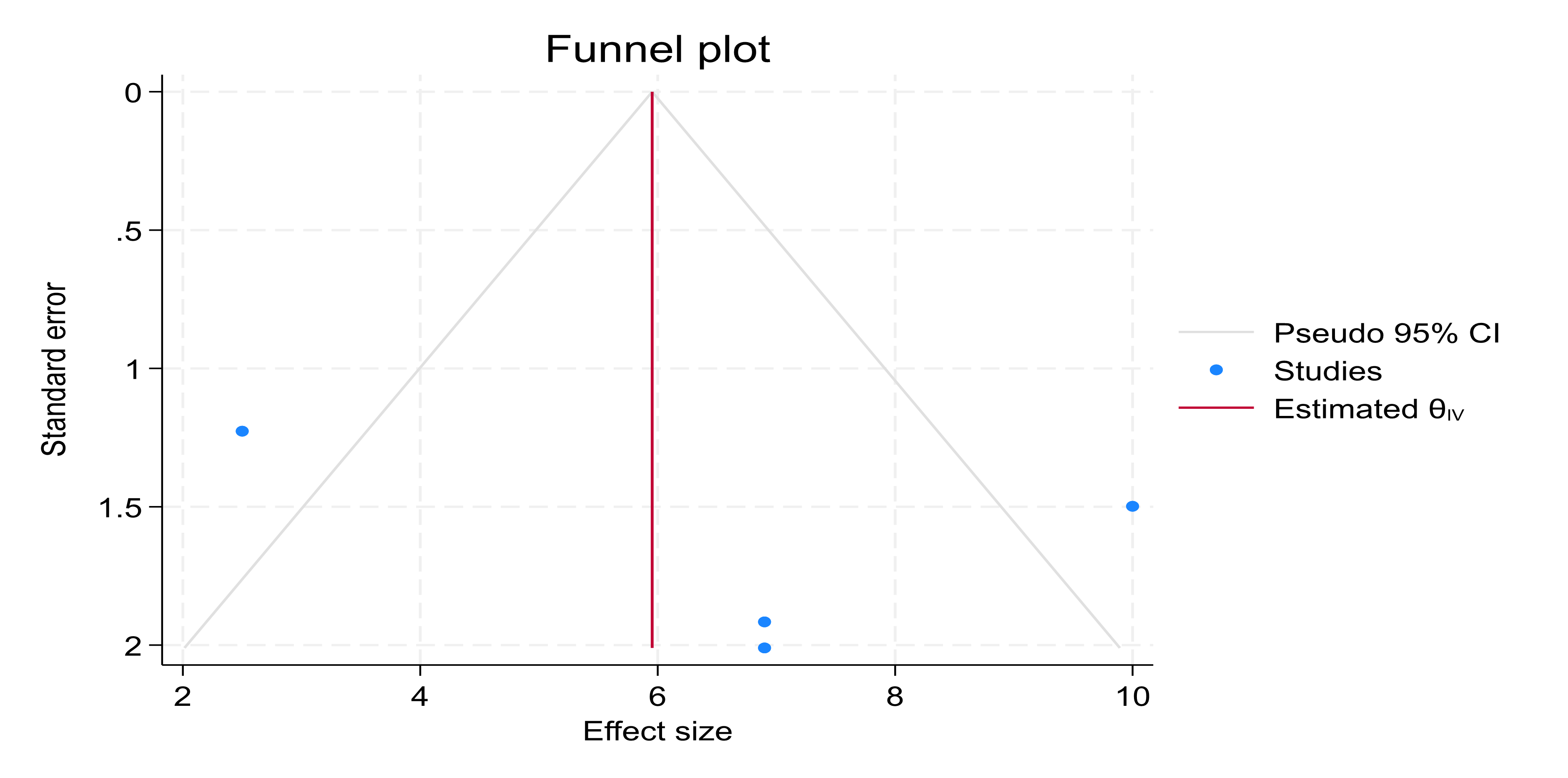
*

Supplementary Figure 3. Funnel plot analysis to assess potential publication bias for mortality(n=4)


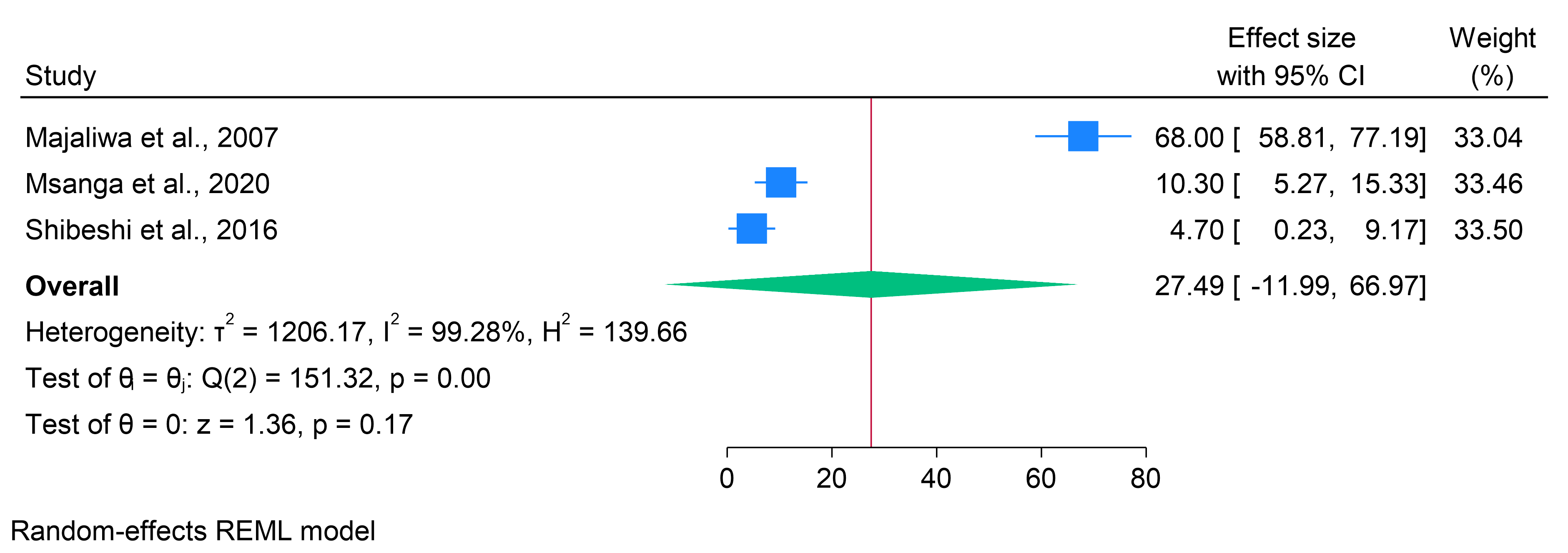


Supplementary Figure 4. Forest plot showing the pooled proportion of retinopathy among children and adolescents with T1DM (n=3).


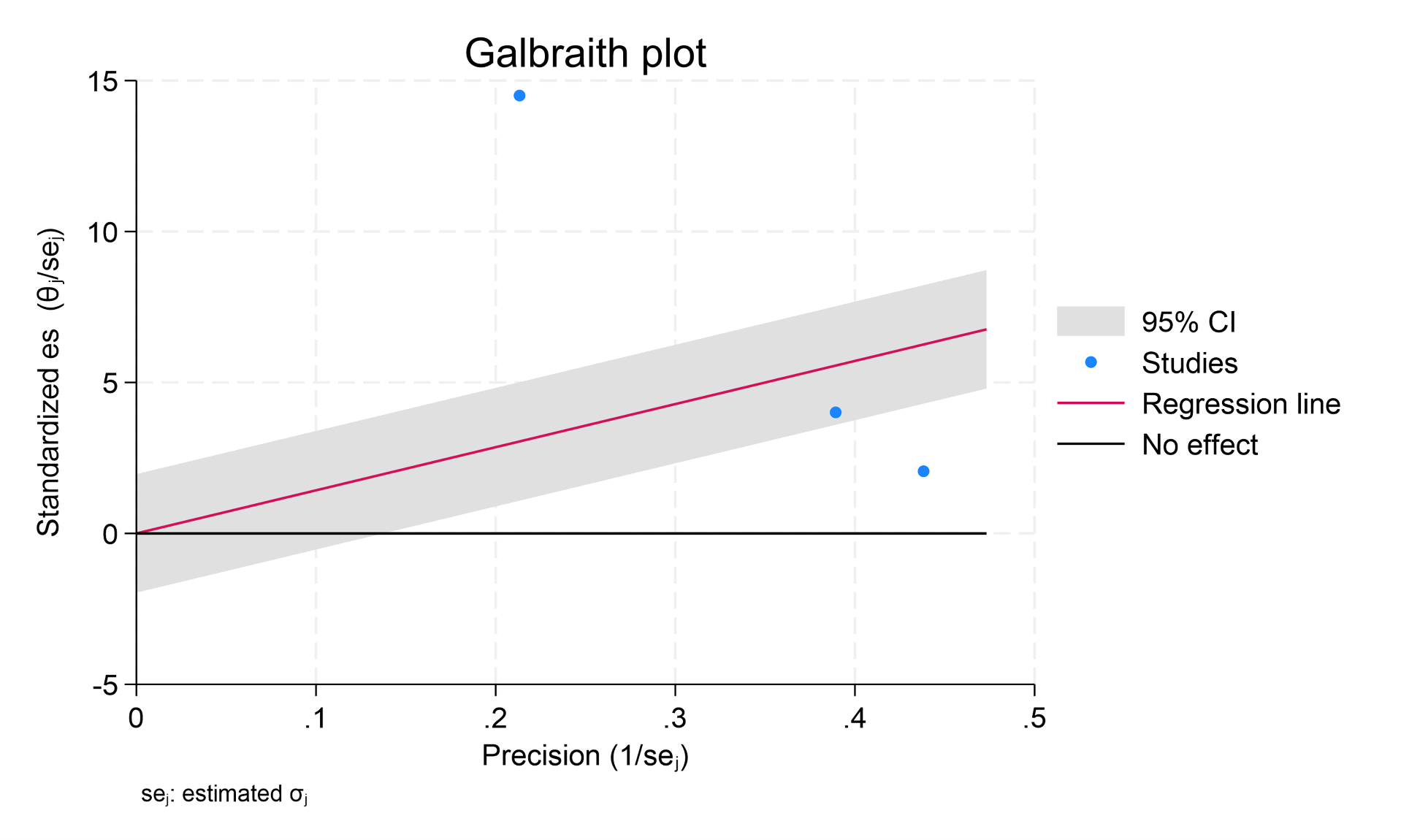


Supplementary Figure 5. Galbraith plot to investigate the source of heterogeneity of retinopathy (n=3)


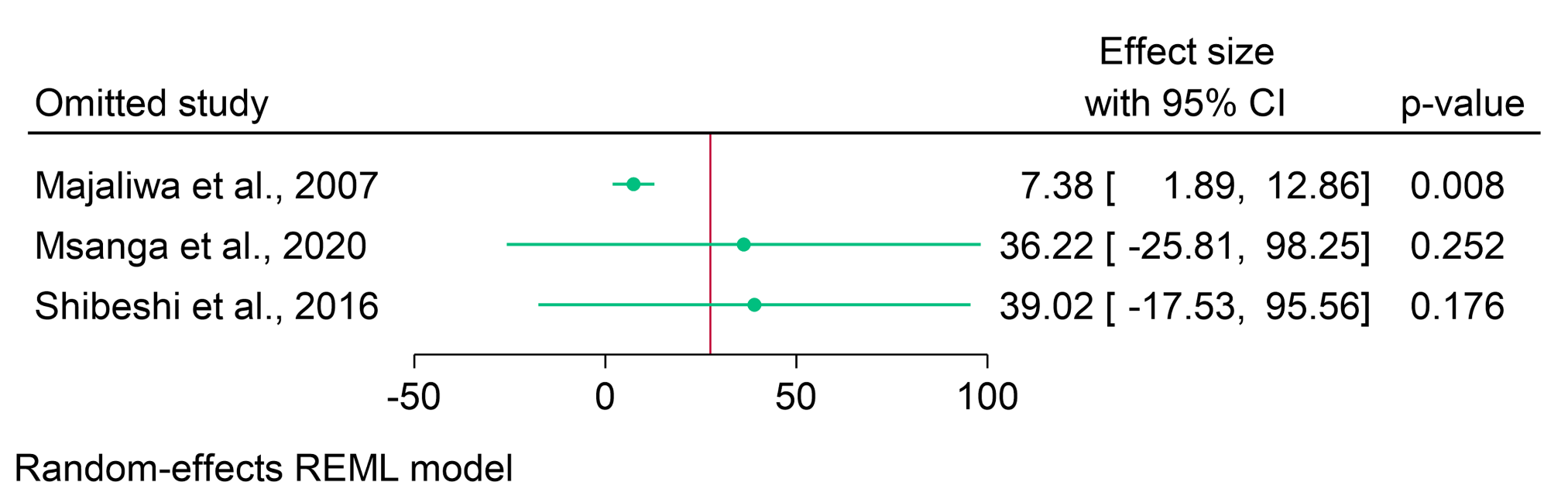


Supplementary Figure 6. Leave-one-out meta-analysis for retinopathy (n=3)


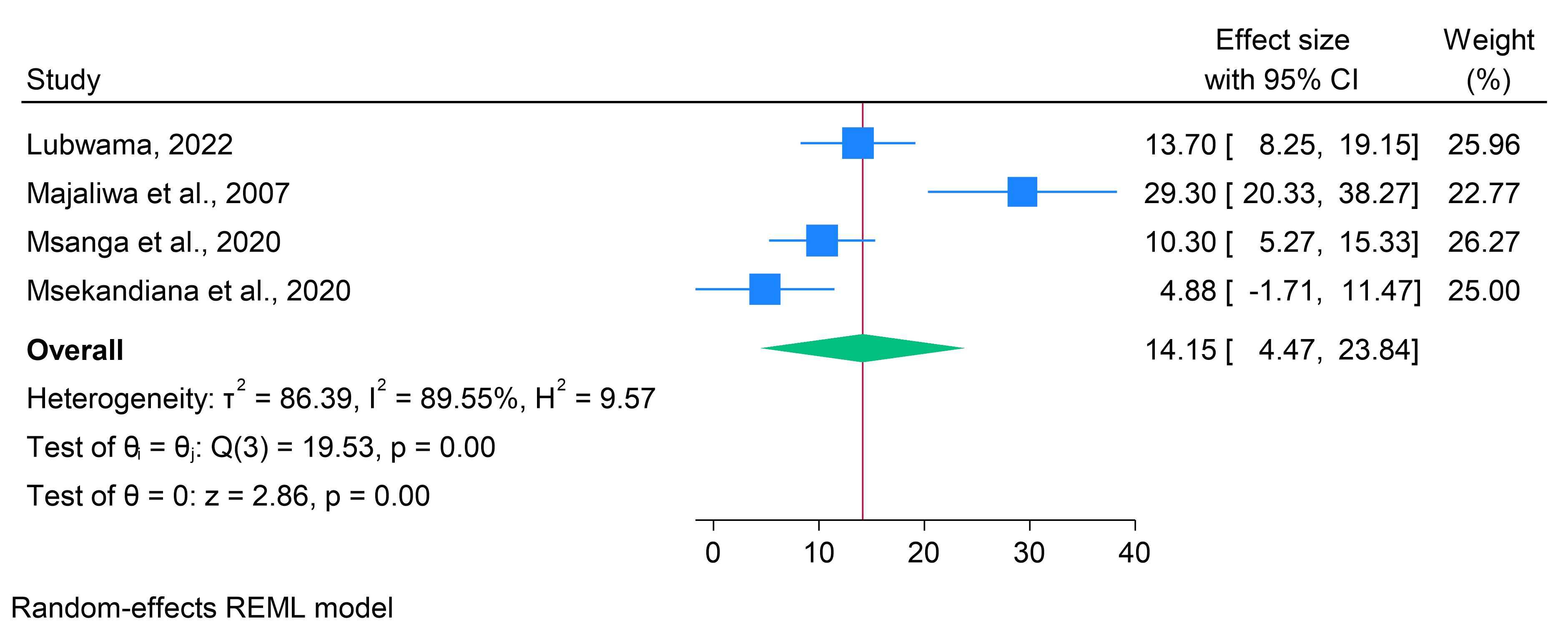


Supplementary Figure 7. Forest plot of the pooled proportion of nephropathy among children and adolescents with T1DM (n=3).


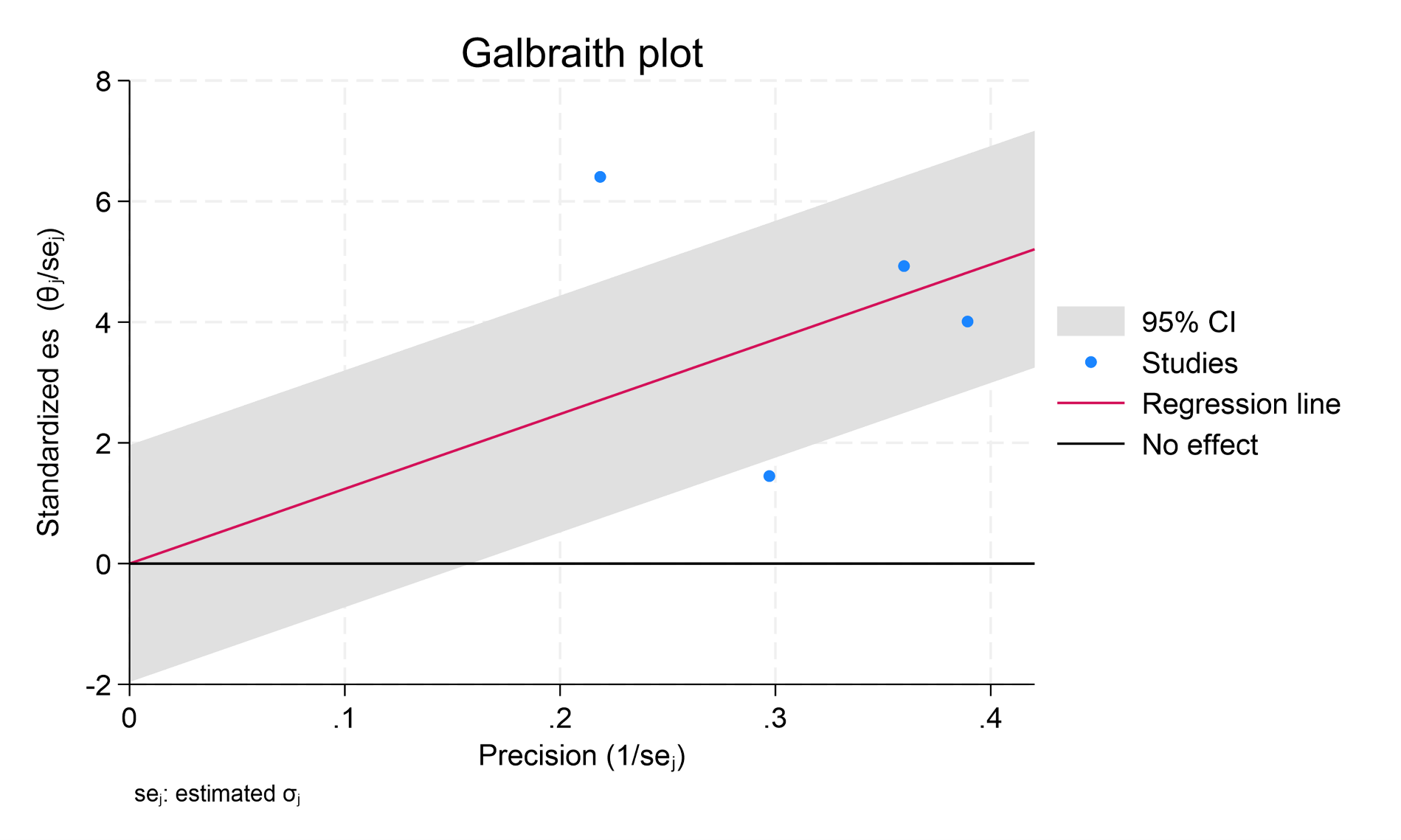


Supplementary Figure 8. Galbraith plot to investigate the source of heterogeneity of nephropathy(n=4).


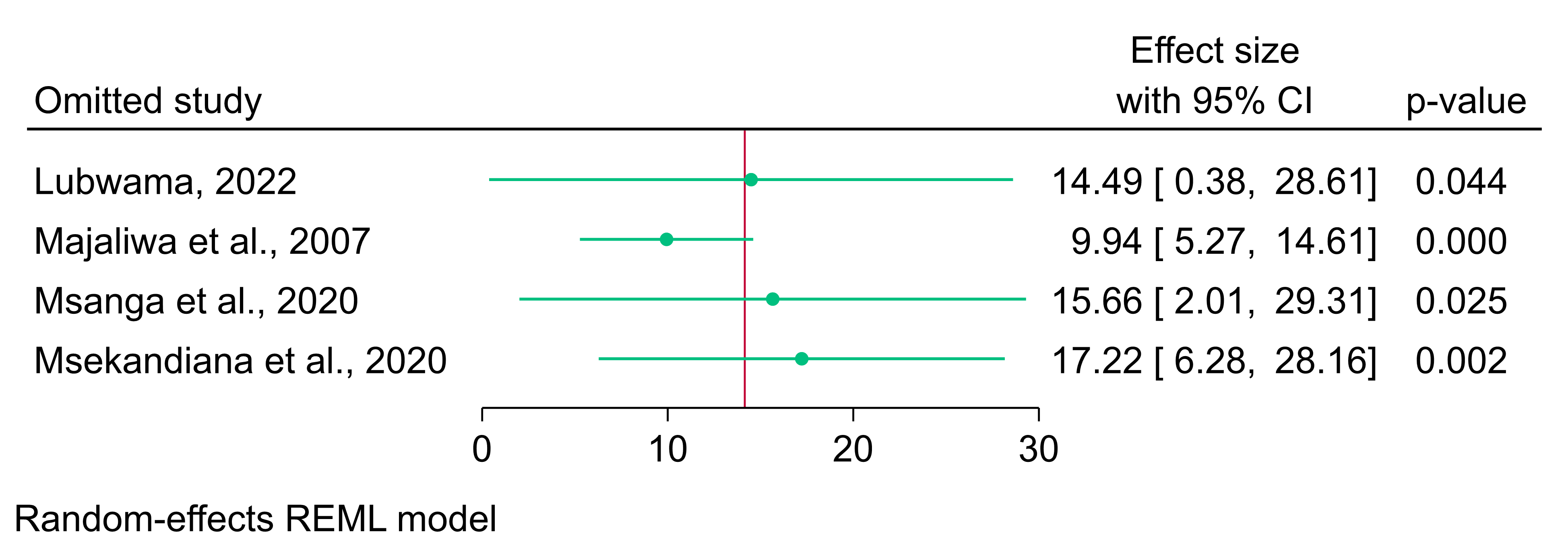


Supplementary Figure 9. leave-one-out meta-analysis for nephropathy(n=4)
